# Using old fields for new purposes: ecosystem service outcomes of restoring marginal agricultural land to forests

**DOI:** 10.1007/s10980-025-02121-0

**Published:** 2025-07-01

**Authors:** Catherine Destrempes, Jesse T. Rieb, John Clark, Gabriela María Torchio, Brian Robinson, Monique Poulin, Elena M. Bennett

**Affiliations:** 1 Department of Natural Resource Sciences, Faculty of Agricultural and Environmental Sciences, McGill University, 21111 Lakeshore Road, Ste. Anne de Bellevue, Québec, H9X 3V9 Canada; 2 Department of Geography, McGill University, 805 Sherbrooke Street West, Montreal, QC H3A 0B9 Canada; 3 Xylem Geospatial Consulting, Ayer, MA 01432 USA; 4 Département de Phytologie, Faculté Des Sciences de l’Agriculture et de l’Alimentation, Université Laval, 2425 Rue de L’Agriculture, Québec, Québec G1V 0A6 Canada; 5 Bieler School of Environment, McGill University, 21111 Lakeshore Road, Ste. Anne de Bellevue, Québec, H9X 3V9 Canada; 6 Quebec Centre for Biodiversity Science, McGill University, 1205 Dr. Penfield Avenue, Montreal, QC H3A 1B1 Canada

**Keywords:** Ecosystem services, Nature based-solutions, Restoration, Modeling, Landscape planning, COP15

## Abstract

**Context:**

Human activities, particularly intensive agriculture, have caused significant environmental degradation, reduced ecosystem diversity, and increased vulnerability to global change. Recent international policies, such as the Global Biodiversity Framework’s 30 × 30 target, advocate for nature-based solutions (NbS) such as ecological restoration to address these impacts. In agricultural landscapes, however, there are concerns that restoration may impact food production.

**Objectives:**

We investigated how forest restoration, as an NbS, changes the supply of ecosystem services (ES), including potential trade offs with agricultural output. Using the Montérégie region of Québec (southeastern Canada) as a case study, we assessed the influence of restoration extent, spatial configuration, and the original agricultural site conditions on the ES outcomes.

**Methods:**

We modeled ES outcomes for seven ES (crop production, maple syrup production, deer hunting, water quality, carbon storage, pollination, and outdoor recreation) under nine scenarios, which varied by total amount of the landscape restored (3.3%, 10.8%, 30%) and initial conditions of the agricultural fields restored (randomly selected, degraded agricultural field, or abandoned agricultural field).

**Results:**

Our findings indicate that increasing the amount of land restored enhances provision of most ES, though improvement varied by service. The initial condition of restored sites minimally influences ES outcomes. However, the spatial pattern of restoration plays a significant role in determining ES delivery, as restored sites enhance most ES through spillover effects up to 500 m.

**Conclusion:**

This study underscores the potential for combining landscape ecology approaches and ES tools to forecast NbS outcomes and inform landscape planning.

**Supplementary Information:**

The online version contains supplementary material available at 10.1007/s10980-025-02121-0.

## Introduction

Human activities are exerting increasing pressure on the environment, leading to significant environmental and social challenges. There is a growing interest in finding sustainable ways to address these challenges by leveraging natural processes. Nature-based solutions (NbS) use ecological processes to address societal and environmental challenges while aiming to provide multiple benefits, such as maintaining biodiversity and enhancing human well-being (Keesstra et al. 2018; Calliari et al. 2019; Hanson et al. 2020). While NbS encompass multiple domains, ecological restoration stands out as a key approach to tackling some of these pressing environmental issues (Chang et al. 2024).

In recent years, NbS have gained significant traction in policy circles and agendas (Seddon et al. 2021). At the recent 2022 UN Convention on Biological Diversity 15th Conference of the Parties (COP15), for example, countries set an ambitious target of restoring 30% of their degraded areas by 2030, highlighting NbS as a means to achieve societal and environmental benefits while meeting international targets (Calliari et al. 2022; Convention on Biological Diversity 2022). This policy emphasis has sparked a surge in research focused on evaluating the benefits of NbS, especially in the context of ecological restoration of degraded habitats.

Studies evaluating the multiple benefits delivered by NbS are increasing (e.g., Chausson et al. (2020); Debele et al. (2023)), but they remain scattered, with diverse methodologies limiting comparability (Dumitru et al. 2020; Seddon et al. 2021). Evaluating changes in ecosystem services (ES)—the benefits people obtain from ecosystems—are one way to assess the co-impacts of NbS implementation (Millennium Ecosystem Assessment (MA) 2005; Hekrle 2022; Fang et al. 2024). ES science has a long history of exploring synergies and trade-offs, offering a promising approach to evaluating the social-ecological benefits of NbS (Bennett et al. 2009; Cohen-Shacham et al. 2019; Dumitru et al. 2020). While studies on NbS and their impact on ES are growing, much of the literature remains focused on concepts, guidelines, and performance. Despite the global interest in NbS, such as ecological restoration, there is limited attention to how restoration actions impact ES supply (Bullock et al. 2011; von Holle et al. 2020; Fu 2023; Fang et al. 2024). Furthermore, most studies narrowly examine on-site outcomes, overlooking broader spatial spillovers and the influence of surrounding land use on NbS effectiveness (Sowińska-Świerkosz and García 2021; Calliari et al. 2022; Debele et al. 2023; Sowińska-Świerkosz et al. 2024).

Given these gaps, addressing the implications of restoration as an NbS on the broader landscape is becoming increasingly critical, especially as global commitments to NbS continue to grow. Countries have pledged to dedicate one billion hectares to NbS and nearly all countries have committed to using NbS for climate mitigation or biodiversity protection through their Nationally Determined Contributions (NDCs) or commitment to the COP15 targets (Dooley et al. 2022; Seddon 2022; Convention on Biological Diversity 2022). However, key questions remain about which landscapes to restore, to what extent, and in what spatial patterns to optimize benefits for people and nature. One type of area with potential for such restoration is less productive agricultural land. Estimates suggest 385–472 million hectares of agricultural land are abandoned and 1–6 billion hectares are degraded (Campbell et al. 2008; Gibbs and Salmon 2015). Restoring converted lands, such as crop fields, is expected to yield the greatest benefits for carbon and biodiversity if converted to forests or grasslands (Currie et al. 2023). By targeting degraded and abandoned crop lands and converting them to forests, countries might meet their commitments while enhancing ES, potentially without sacrificing crop production.

However, previous studies warn that poorly planned restoration can negatively impact biodiversity and landscape health (Di Sacco et al. 2021). This raises critical questions: What benefits could restoration of agricultural fields provide, and how might initial landscape conditions (land use at the restoration site, surrounding land use) affect the benefits generated? Would the location and extent of restoration influence these outcomes? To explore these questions, we assessed the potential benefits and trade offs of several restoration scenarios.

Our study models the ES impacts of restoring crop land to forest in agricultural landscapes as an NbS. We selected the Montérégie region of Québec, Canada, as a case study for this research due to its rich data on ES and its similarity to other intensive agricultural landscapes striving to meet COP15 goals (Simelton et al. 2021; Debele et al. 2023). Using a landscape-wide restoration approach, we modeled the effectiveness of forest restoration on selected agricultural fields—whether randomly chosen, abandoned, or degraded—to achieve various levels (3.3%, 10.8%, or 30%) of the COP15 Target 2 goal which aims to restore 30% of degraded land by 2030. Through these nine restoration scenarios, compared to a baseline scenario (0% restoration), we quantified the impact on seven ES: crop production, maple syrup production, deer hunting, water quality regulation, above- and below-ground carbon storage, pollination and outdoor recreation.

Overall, our research has three primary objectives: first, to assess the impact of restoring agricultural fields with varying initial conditions—random, degraded, or abandoned—on the supply of ES; second, to evaluate how varying extents of restoration in agricultural areas, corresponding to different levels of the COP15 goal, impact the supply of multiple ES based on spatial patterns at the landscape scale; and third, to evaluate the extent of the impact that NbS generate within and beyond the limits of the restored sites.

## Methods

We quantified the ES outcomes of restoring agricultural lands into forest in different spatial patterns across the Montérégie region in Québec. We created nine restoration scenarios using three ways to choose restoration sites (agricultural land that is abandoned, agricultural land that is degraded, or randomly selected agricultural land) and varying the amount of land restored (3.3%, 10.8%, or 30% of the total agricultural land area of the region). We compared these to a baseline scenario (0% restoration), for a total of 10 scenarios (Table 1). Forest restoration, our selected NbS in this study, involves converting agricultural land to mature forest typical of the region, without distinguishing forest types. The study assumes equilibrium forest conditions. Changes in local climate are not explicitly taken into account.

**Fig. 1:**
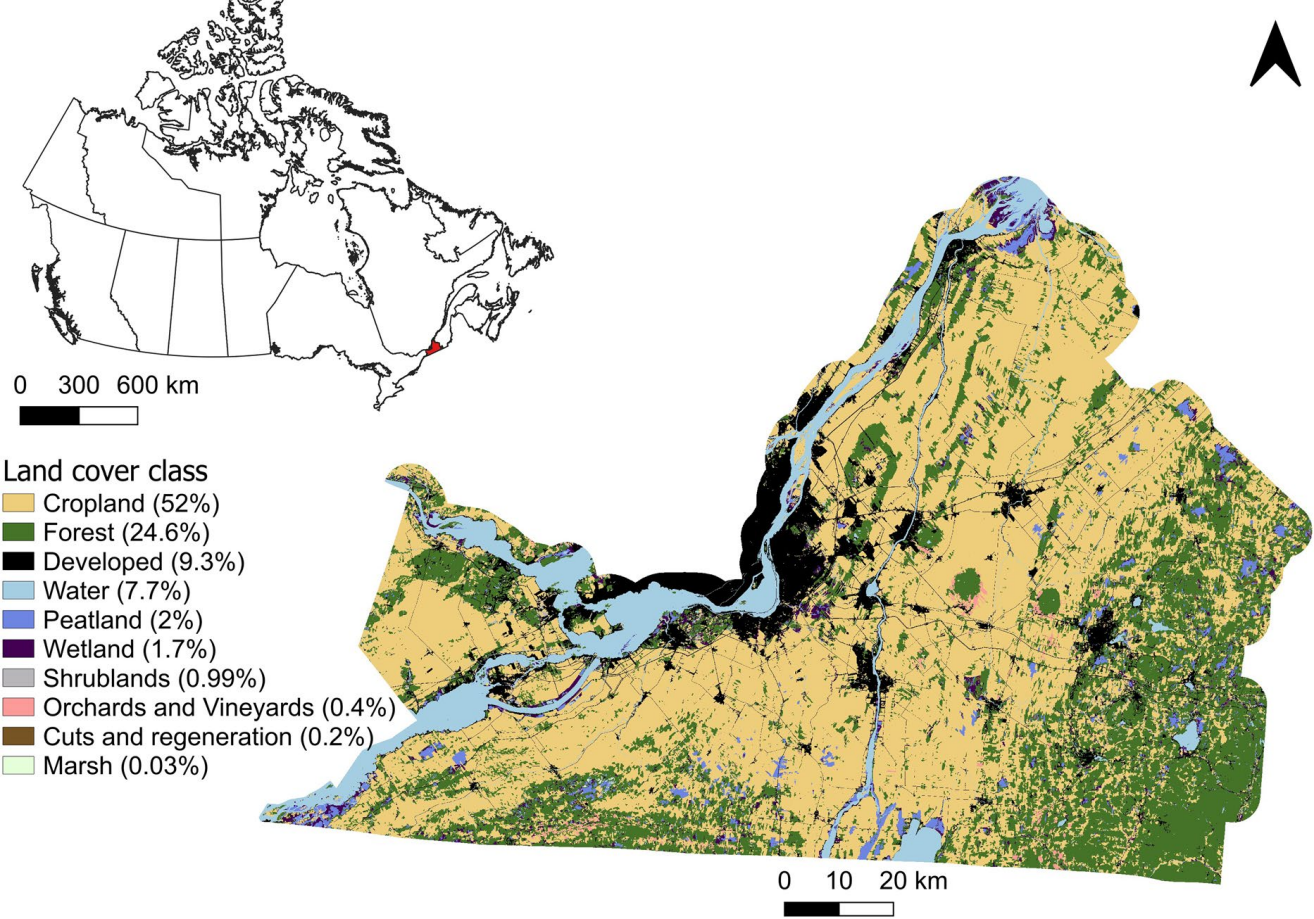
Top left, map of Canada with the location of our study region in red. Bottom right, land cover map of our study region, divided into 10 classes representing the main cover present in each (30 × 30 m) pixel. Overall coverage is indicated in parentheses next to each class (Ministère de l’Environnement, Lutte contre les changements climatiques, Faune et Parcs 2015 (MELCCFP))

**Table 1:**
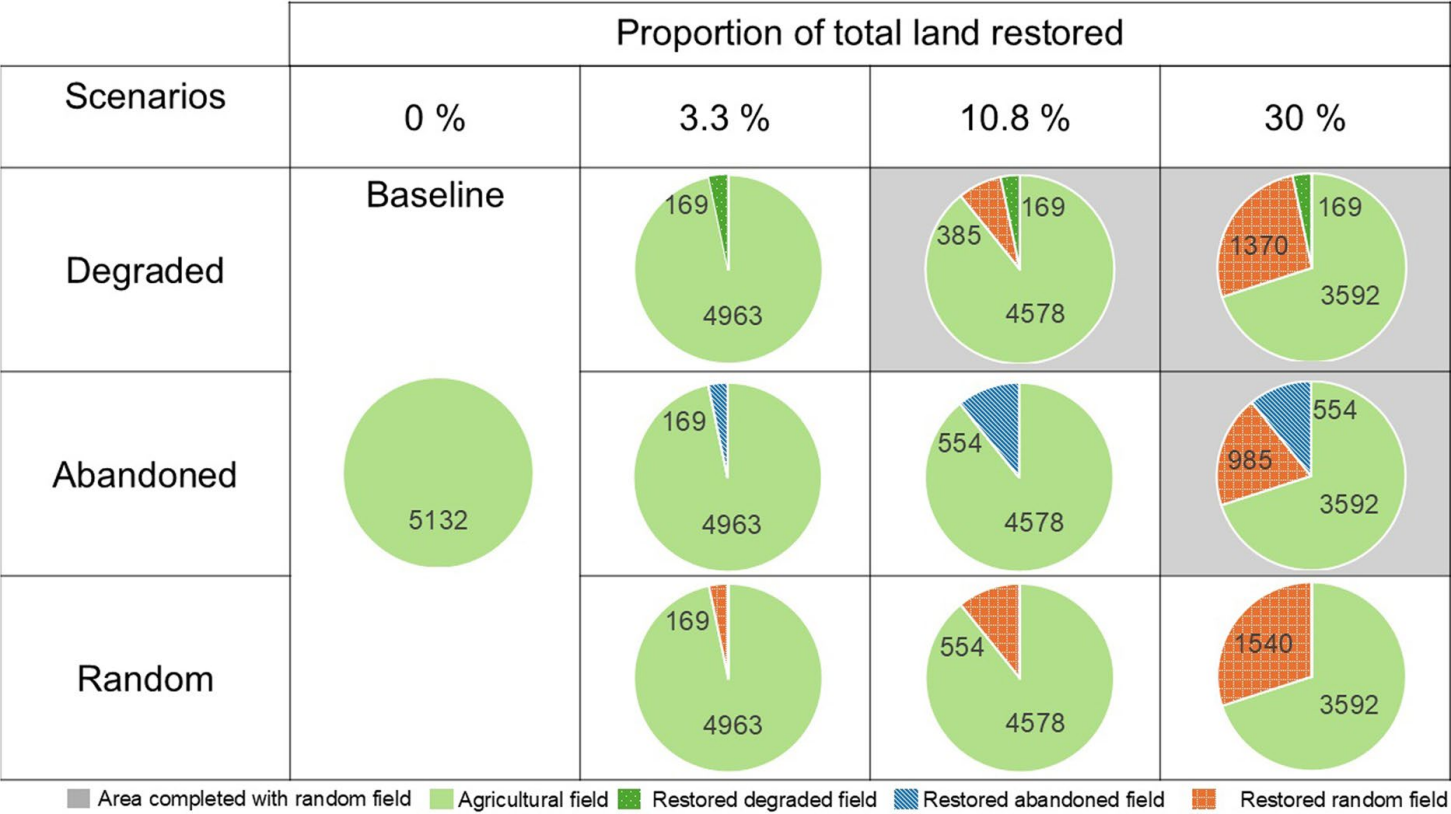
Proportion of agricultural land restored in each of the 10 scenarios compared. Each pie chart shows the total area (5132 km^2^) in the Montérégie classified as agriculture and details which type of field was chosen to be restored to forest in each scenario (exact areas in km^2^ are indicated). The three scenarios with grey backgrounds also include randomly selected agricultural fields (not abandoned or degraded) to meet the required restoration percentage

### Study area

Our study area, the agriculturally intensive Montérégie region, covers around 10,000 km^2^ in southern Québec, Canada, (Fig. 1) and has some of the highest biodiversity potential in the province (Tardif et al. 2005; Ministère de l’Agriculture, des Pêcheries et de l’Alimentation 2014; Mitchell et al. 2015; Currie et al. 2023). Although administrative changes led to a new boundary for the region in 2021, we used the pre-2021 boundary for our study to make use of the extensive ES data available for the older administrative region (Raudsepp-Hearne et al. 2010; Mitchell et al. 2015; Rieb and Bennett 2020; Institut de la statistique du Québec 2021). The Montérégie has highly fertile soil, which explains why such a large number of its forests have been converted to agriculture over time (Bélanger and Grenier 2002; 2014). By 2018, the Montérégie had only 17% forest cover, while 54% of its land was used for agriculture (Communauté métropolitaine de Montréal 2012; ECCC and MDDELCC 2018). However, not all land classified as agricultural is actively used for growing crops (Municipalités régionales de comté de La Vallée-du-Richelieu 2016), and restoration of less productive agricultural land may be an opportunity to increase forest cover while minimizing the impact on agricultural productivity.

### Scenario creation

To create our 10 restoration scenarios, we began by identifying the agricultural land, degraded agricultural fields, and abandoned agricultural fields in the Montérégie, as outlined below. We then determined the total area to be restored based on COP15 guidelines, which targeted the restoration of 30% of all degraded land, including transformed ecosystems such as agricultural land (United Nations Environment Program 2022; Convention on Biological Diversity 2023). Consequently, our maximum restoration target was set at 30%, but we also explored reduced targets based on the proportion of agricultural land identified as abandoned (10.8%, 554 ha) and degraded (3.3%, 169 ha) (see Fig. S4 for abandoned and degraded field locations). For scenarios in which we do not have enough degraded or abandon land to meet the 10.8% or 30% targets, additional fields were selected at random (Table 1). For simplicity, we rounded down the total amounts in our scenarios, though we identified 558.45 km^2^ (10.88%) of abandoned land and 170.62 km^2^ (3.32%) of degraded land in the Montérégie.

#### Identifying random fields

To select random samples of fields for restoration, we used a dataset developed by La Financière agricole du Québec (FADQ) (2016) that included all crop fields in Québec that were insured in any year between 2003 and 2014. We clipped this dataset to include only fields within the Montérégie. We then randomly selected fields from this dataset until we had reached 3.3%, 10.8%, or 30% of the agricultural land in the Montérégie.

#### Identifying degraded fields

The literature on degraded agricultural fields presented a variety of criteria for identifying degradation (Gibbs and Salmon 2015). We adopted crop productivity as measured by the Normalized Difference Vegetation Index (NDVI) as our indicator for degradation. NDVI is widely used to monitor land degradation due to its capacity to assess vegetation condition (Gibbs and Salmon 2015; Rieb and Bennett 2020). Following the methodology outlined by Torchio et al. (2024), we employed Google Earth Engine and Landsat 5, 7, and 8 satellite images to calculate NDVI. We processed and filtered Landsat images from June to September over a 21-year period (2000–2021), resulting in a merged NDVI image for each year. To mitigate edge effects on NDVI measurements, we automated the creation of a 25 m buffer around each field boundaries in the FADQ (2016) (Montandon and Small 2008). We then performed a linear regression plotting year against NDVI values for each pixel on the map to determine slopes and p-values. All pixels showing significant decrease in NDVI over the 21-year period were considered to be declining in productivity. We considered those fields to be *degraded* if 30% or more of their area was covered by pixels that showed productivity decline over all 21 years. All calculations and analyses were conducted in R, ensuring reproducibility and precision in identifying degraded fields.

#### Identifying abandoned fields

We considered fields to be abandoned if they were unused for five consecutive years, a threshold chosen based on the standard definition of cropland abandonment (Grădinaru et al. 2015; Yin et al. 2020). Abandoned fields were identified by synthesizing three datasets: land cover, forest inventories, and field productivity.

We used land cover data provided by Agriculture and Agri-Food Canada's (AAC), in their Annual Crop Inventory (ACI), a 30-m raster time series spanning the years 2011–2015 (AAC 2023). To identify a field as abandoned in our baseline year (2014), we noted the presence of any land cover class that could indicate land abandonment (e.g., pixels coded as shrubland, exposed land, fallow land, or forest). To differentiate between land left fallow for temporary rest periods and fields that were genuinely abandoned, only pixels that were consistently classified as shrubland, exposed land, or fallow across the five-year period were selected. To aggregate from the pixel scale to agricultural fields, any polygon from the FADQ that contained abandoned pixels was identified as abandoned.

We used the forest inventory data provided by the Ministère des ressources naturelles et des forêts du Québec (MRNF) (2016) as our second dataset. These shapefile-based inventories reflect landscape evolution at decadal intervals, with an emphasis on forest cover and disturbance. The 1991–2003 and 2001–2018 inventories provided an approximately 20-year period to identify abandoned fields, aligning with the period previously use to identifying degraded fields (Gouvernement du Québec 2024). Fields classified as agriculture in 1991–2003, but as "friche" (fallow) or "tending to forest vegetation" in 2001–2018, were identified as abandoned fields.

The last dataset we used to identify abandoned fields was the field productivity data provided by La Financière agricole du Québec (2016), census data, which contains polygons for each insured field in the region. Fields were considered abandoned if no production was reported from 2010 to 2014, inclusive. Additionally, the census began designating abandoned filed has "old fields" in 2013. Polygons classified as "old fields" in both 2013 and 2014 were also identified as abandoned.

If a field was identified as abandoned in any of the three datasets, it was considered abandoned in our study. Therefore, we combined these three maps into a single composite of all abandoned fields in the Montérégie. Overlapping and redundant polygons introduced by the merge operation were eliminated using the 'delete duplicate geometry' and 'topology checker panel' tools in QGIS (QGIS Development Team 2024).

## Modeling ecosystem services

We modeled seven ecosystem services (ES) in this study: crop production, maple syrup production, deer hunting, water quality regulation, above- and below-ground carbon storage, pollination, and outdoor recreation. These services were selected based on their importance to the region and their prior use in local studies, ensuring data availability (Mitchell et al. 2015; Renard et al. 2015; Rieb and Bennett 2020). We used three distinct modeling approaches to quantify ES supply across our scenarios: 1) NDVI trends for crop production (Rieb and Bennett 2020; Statistics Canada 2023); 2) the InVEST (3.13) platform for water quality regulation, carbon storage, and crop pollination (Spawn et al. 2020; Rieb and Bennett 2020; Sothe et al. 2022; Zhang et al. 2024); and 3) Maxent (3.4.4) to quantify white-tailed deer hunting, outdoor recreational potential, and maple syrup production potential (Phillips et al. 2006). Detailed plots presenting model validation and quality as well as a complete table summarizing each variable used to create our ES models are provided in the Supplementary information (Table S6).

### Crop production (NDVI)

We used NDVI to quantify agricultural crop production for each scenario. NDVI measured over the growing season is a reliable and widely used indicator of crop production (Bédard et al. 2010; Statistics Canada 2023). We adopted the map by Rieb and Bennett (2020) of NDVI-based crop production (kg/ha/year) in 2014 as our baseline for the 0% restoration scenario, representing theoretical yields, as actual yield maps were unavailable at resolutions equal to or finer than 30 m. Due to methodological differences, Rieb and Bennett (2020) measured NDVI for all fields, regardless of abandonment status. Therefore, the raw data used for the baseline map showed positive theoretical yields for some fields that we classified as abandoned. To ensure consistency between all the scenarios, we adjusted the baseline map to give any abandoned fields a yield value of zero (see S1 for more information).

### Water quality regulation, carbon storage, and pollination (InVEST)

We assessed water quality by characterizing it as the total phosphorus exported from the landscape into the watershed. This was calculated using the Integrated Valuation of Ecosystem Services and Tradeoffs (InVEST 3.13) platform (Natural Capital Project 2024), which models spatially explicit flows of water and nutrients across a landscape. We first quantified the annual water runoff from each pixel within our study area using InVEST’s Annual Water Yield model, with region-specific parameters adopted from Rieb & Bennett (2020). Water yield was then used as an input to the InVEST Nutrient Delivery Ratio model to quantify nutrient runoff, following Qiu & Turner (2013). Land Use and Land Cover (LULC), which varied for each restoration scenario, were used to determine nutrient sources across the landscape and nutrient retention capacity per pixel. This generated a map of phosphorus export per pixel in kg/ha/year for each scenario (see value used in table S1, S2 and S6) (Natural Capital Project 2024). Finally, to make interpretation more intuitive, values were multiplied by − 1, with negative values representing lower water quality and positive values higher (Rieb and Bennett 2020).

We used the InVEST 3.14.1 platform to assess carbon (C) storage based on LULC and three additional layers: mapped soil C values (Sothe et al. 2022), above-ground carbon, and below-ground carbon (Spawn et al. 2020). These layers were resampled to 30-m resolution to align with our LULC data. We extracted the average C stored (t/ha) for each LULC class across all three maps (above-ground, below-ground, and soil). These averages were then used as the lookup table required to run the InVEST C model (see supplementary Table S3). We then used this table and the InVEST model to generate a raster map representing the total carbon stored across the entire landscape in each of our ten scenarios (Natural Capital Project 2024).

We used the crop pollination module in InVEST 3.13 to assess pollination in each of our scenarios (Natural Capital Project 2024). This model measures nesting sites and food supply for pollinators by calculating the suitability of the landscape to provide these resources to pollinators on a scale from zero (not suitable) to one (highly suitable). The model requires three inputs: 1) a guild table, which provides information on each bee species of interest such as their flight range, feeding, and nesting site needs; 2) an LULC map of the region of interest; 3) and a biophysical table illustrating the suitability of different land use types for pollinators (see supplementary Table S4-5 for more detail) (Natural Capital Project 2024). Our focus was primarily on a single wild species, the bumble bee (*Bombus* spp.), due to the availability of pertinent information (Zhang et al. 2024). We applied a buffer to mitigate edge effects, but due to the absence of U.S. land cover data, the buffer did not extend across the Canada-USA border. Using these three inputs, we calculated pollinator abundance based on the floral resource available at each pixel and the accessibility of these pixels to pollinators. This provided an estimate of the total abundance of pollinators in summer across the landscape, for each restoration scenario (Natural Capital Project 2024).

### Outdoor recreation, white-tailed deer hunting and maple syrup production (Maxent)

We used Maxent (version 3.4.4) to model the suitability to provide outdoor recreation, white-tailed deer hunting, and maple syrup production (Phillips et al. 2024). Maxent is a widely used tool for species distribution modeling but is also effective for mapping the suitability of human activities and ES (Seda Arslan et al. 2021; Goodbody et al. 2021; Aouinti et al. 2022). Maxent works with presence-only data, overcoming the challenge of not having absence data, as was the case in our study (Elith et al. 2006). Maxent is also able to integrate landscape metrics when modelling ES suitability scenarios (Phillips et al. 2024).

Each model created using Maxent underwent a stepwise selection process to identify the most appropriate environmental variables for each service. Once developed, these models were applied to all scenarios in this study to generate maps predicting the suitability of each service under each scenario. While the specifics are not discussed here, you can refer to the Supplementary information (Tables S6 and S1 subsection 1.3) for detailed explanations of Maxent, the stepwise process, and the recovery and processing of each variable. Below, we provide a brief overview of the selected variables and presence points used for each ES model generated through Maxent.

#### Outdoor recreation

To model the suitability for outdoor recreation across scenarios using Maxent, we first needed an indicator of outdoor recreation to serve as our response variable. For this, we used geotagged photographs sourced from Flickr, collected between 2000 and 2015, excluding urban areas (Rieb and Bennett 2020). Flickr was known as a valuable indicator for identifying sites frequented for outdoor recreation and for tracking broader outdoor recreation trends due to its extensive user base and daily uploads during the time period studied (Rieb and Bennett 2020; Seda Arslan et al. 2021; Goodbody et al. 2021).

Our stepwise selection resulted in a model that used eight environmental variables to predict outdoor recreation for each of our scenarios (see Supplementary information S1 subsection 1.3, for stepwise selection process). Only one climate variable was selected: precipitation during the wettest month (Deb et al. 2020; Goodbody et al. 2021). For landscape metrics, distance to any edge, as well as distance to forest, roads, trails, recreation sites, and water bodies, LULC, and patch size remained in the final model.

#### White-tailed deer hunting

We used Maxent to map the suitability for white-tailed deer hunting in each scenario following Rutten et al. (2019) by extracting data on white-tailed deer kills reported by hunters to the Ministry of Forests Fauna and Parks Québec between 2008 and 2012 (Renard et al. 2015). Each deer killed represented a presence point for the purpose of Maxent.

We applied the same stepwise selection process as for outdoor recreation previously and identified eight environmental variables good for predicting hunting potential (see Supplementary information S1). These included three climate variables—isothermality and precipitation of the wettest month (mm) from the Wordclim database, along with maximum temperature in 2014 (Thornton et al. 2017, p. 3). It also included five landscape metrics—patch size, Shannon diversity index (SHDI), a measure of diversity and evenness of LULC, and three distances to edge metrics—distance to forest, roads and water bodies.

#### Maple syrup production

We extracted the locations of forests with sugar maple trees in the region from the 2001–2018 forest inventories conducted by the MRNF (2016), using the complete point dataset of tree species and locations available. We selected tree species from this database and filtered to keep points representing sugar maples. This became our presence data, representing the suitability for syrup production in Maxent.

Our stepwise selection process revealed that four environmental variables were good predictors for the maple syrup potential. Only one climate variable, the minimum temperature of the coldest month, was found to be a good predictor for sugar maple. Three landscape metrics were found to be good predictors of maple syrup potential: patch size, LULC, and distance to forest edge.

## Statistical analysis

### Correlation analysis

To evaluate potential correlations between ES that might influence the results and interpretation of our scenarios, we conducted a Spearman pairwise correlation analysis using the raster data from our no-restoration scenario (Tomscha & Gergel 2016) (see Supplementary Information Fig. S7 for the correlation output).

### Spatial pattern analysis

Given the extensive dataset of 70 maps (seven ES across 10 scenarios), we restrained our pixel scale examination of spatial pattern variations only to random field scenarios, since some abandoned and degraded scenarios already integrated random field selection to attain our restoration objectives. Other restoration types were assessed visually at the landscape scale, allowing us to identify broader spatial trends and patterns.

#### Buffer analysis

To evaluate the ecological impacts beyond the restored fields, we analyzed surrounding zones by generating buffers around each restored field at multiple distances. For each random scenario, we created five buffer zones at distances of 50 m, 100 m, 250 m, 500 m, and 1000 m from the edges of the restored fields using the previously generated ES supply maps. We summed the ES supply values within each buffer zone, excluding any values from within the restored fields themselves. This allowed us to calculate the ES supply per unit area within each buffer and compare it to baseline conditions without restoration. Through this, we could identify any changes in ES supply due to restoration and evaluated if and how far restoration benefits extended into surrounding areas.

#### Comparison of restoration scenarios to the baseline

To pinpoint and assess the precise locations affected by restoration, we conducted a pixel-level analysis at a resolution of 30 m x 30 m, comparing each scenario against the baseline. This detailed examination focused exclusively on random scenarios to maintain consistency given time constraint. This high-resolution approach enabled us to capture fine-scale spatial heterogeneity and assess localized changes in ES supply.

To facilitate meaningful comparisons, we standardized our ES supply values by applying a min–max normalization, ensuring that all services measured ranged between 0 and 1 (Lamy et al. 2016; Li et al. 2019). When performing the standardization, we grouped the scenarios by ES to ensure that the true maximum and minimum value for each ES were used to standardize. All steps were performed using the RStudio platform. Scripts are available upon request.

Overall, several analyses were performed on the data generated from each of our scenarios. However, due to the absence of multiple scenario replicates, which was constrained by long computing times, our ability to assess variability was limited. To address this, we compared three field restoration types, which served as a proxy for replicates. Future studies should incorporate multiple iterations to enhance precision.

## Results

### Ecosystem service supply at baseline

**Fig. 2:**
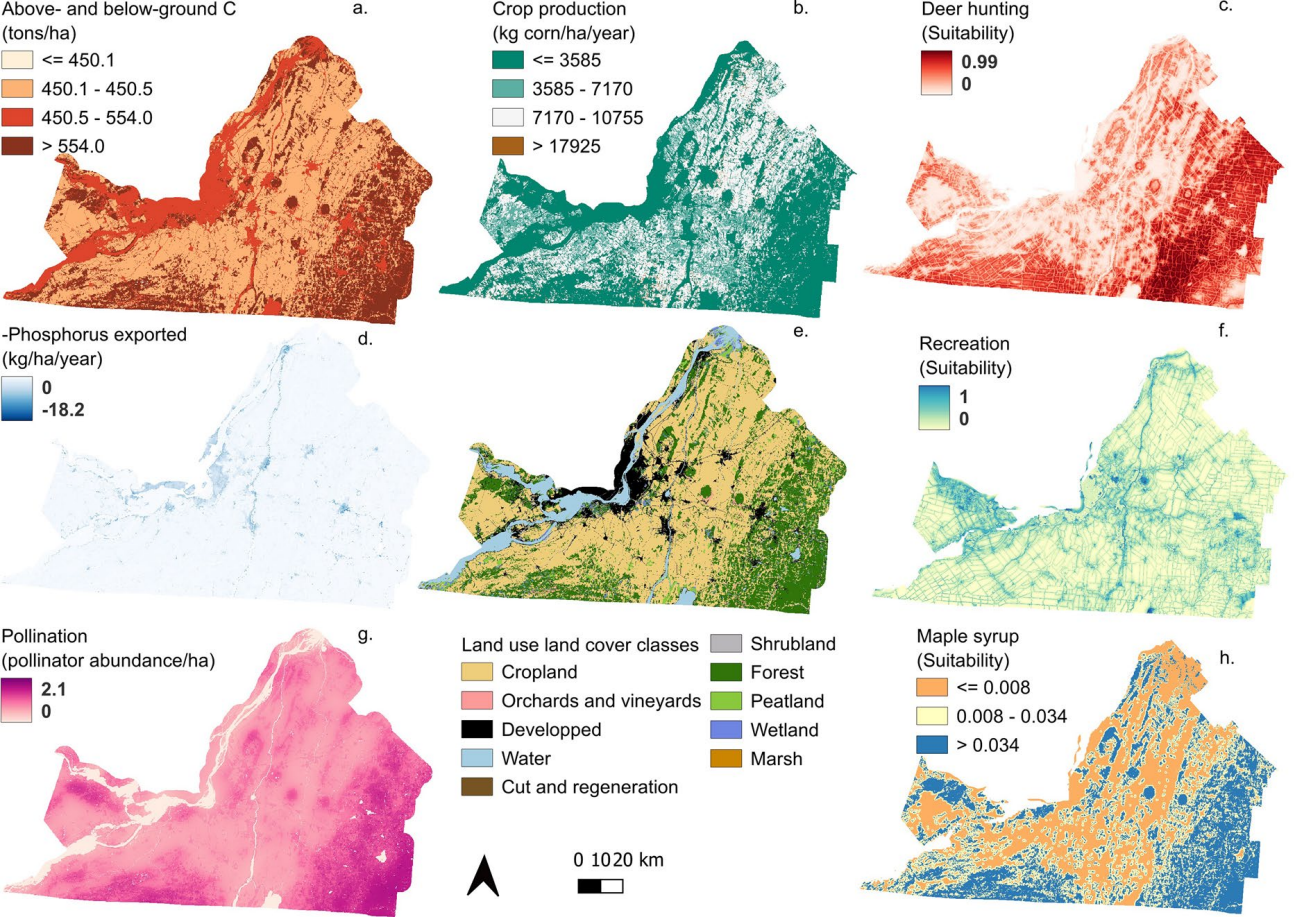
Map of ES supply at baseline (2014) in the Montérégie. The ES depicted are: **a** above- and below-ground carbon storage in tons per hectare of C, **b** crop production (kg corn/ha/year), **c** potential for hunting white-tailed deer (suitability), **d** water quality through—phosphorus exported to the watershed (-kg/ha/year), **e** land use land cover in 2014, **f** outdoor recreation potential (suitability), **g** pollination potential (suitability) and **h** maple syrup production potential (suitability). Maps that depict suitability are ranked from zero (not suitable at all) to one (perfectly suitable)

Baseline conditions provided a reference for the spatial distribution and interactions of ES in the study region. As shown in Fig. 2, ES supply generally aligned spatially with land use and land cover (LULC) categories. For instance, at the landscape level, agricultural field yield was highest for parcels located near natural and forested patches of the landscape and lowest for fields surrounded primarily by other croplands, such as in the central part of the study region where these natural patches are less common. Additionally, pollination potential also positively peaked near these natural patches. Similarly, water quality regulation was less effective in urban areas, reflecting differences in land cover suitability to provide this ES (Fig. 2d). Outdoor recreation, hunting, and maple syrup production showed limited suitability across the region, reflecting a generally low supply of these ES in the landscape. These patterns emphasized the role of LULC in shaping the supply and interactions of ES in this study (Supplementary Fig. S7). At baseline, most ES were uncorrelated, with exceptions such as a positive correlation between maple syrup production and hunting and a weak negative correlation between maple syrup production and agriculture.

### Change in ecosystem services supply across scenarios

#### Impact of initial site condition and percentage of area restored on ES supply

**Fig. 3:**
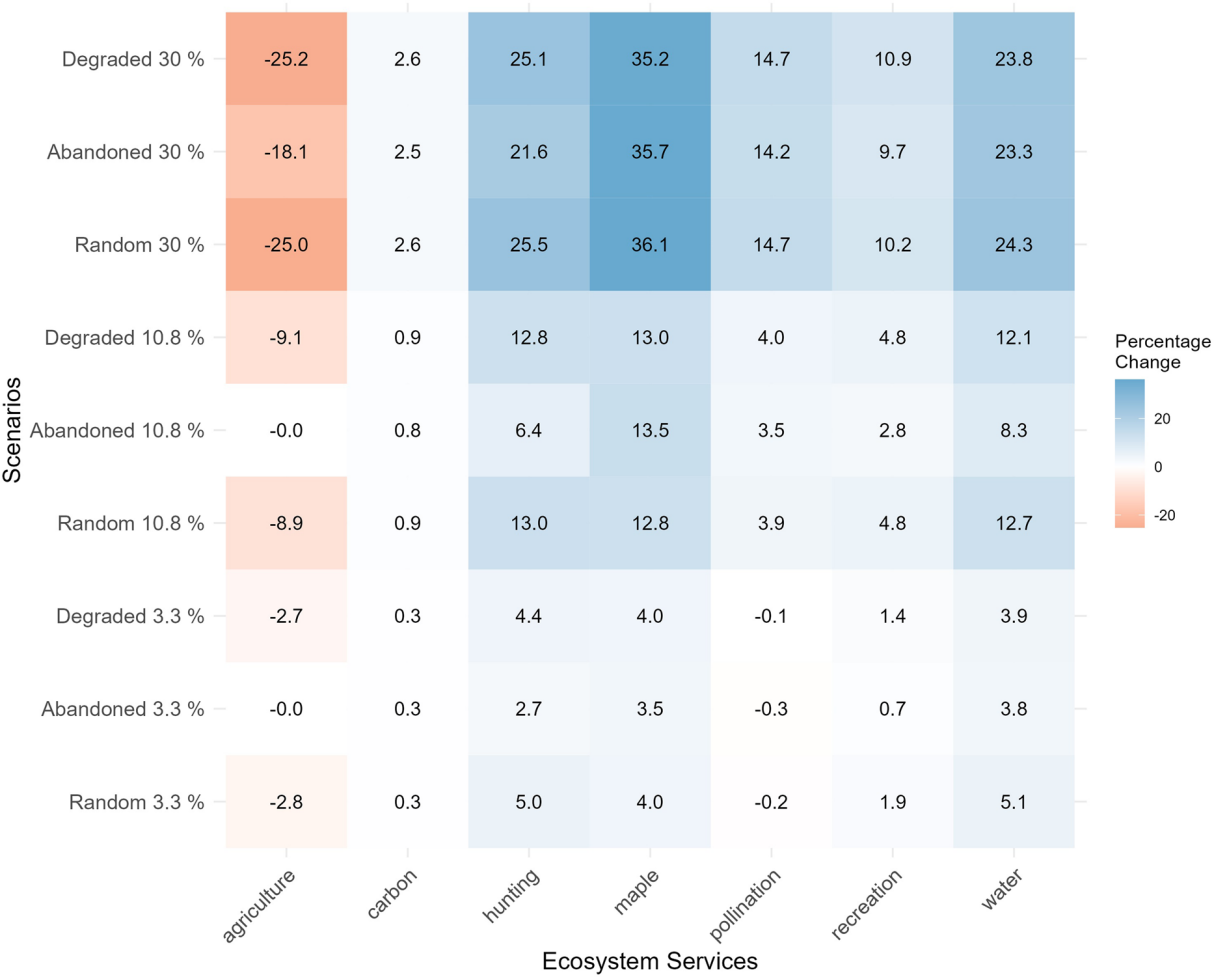
Heatmap of the percentage difference from the baseline ES supply in each scenario. Each row shows a scenario, and each column shows an ES. Orange shows a decrease, and blue shows an increase in ES supply as compared to the baseline

The supply of each ES varies depending on the amount of restoration and the initial condition of the restored fields. The Montérégie has about 5130 km^2^ of agricultural lands, of which 558.45 km^2^ (10.88%) is abandoned, and 170.62 km^2^ (3.32%) has degraded over the 21-year period from 2000 to 2021. Below 10.8% restoration, restoring abandoned fields had no impact on agricultural yield due to the initial lack of productivity in abandoned fields, while restoring degraded or random fields led to agricultural productivity losses (Fig. 3). Despite the minimal impact on agricultural production, the restoration of abandoned fields showed an overall lower ES gain compared to restoring degraded or random fields. Maple syrup production was an exception, being higher in abandoned fields at 10.8% restoration compared to degraded or random fields (Fig. 3).

The initial site condition with the highest ES supply varied across scenarios. For example, hunting and water quality consistently showed higher supply when restoring random fields, while pollination was higher when restoring degraded fields (Fig. 3). As the restoration area increased, most ES supply also increased, regardless of the field type restored, except for agriculture, which decreased in degraded and random fields. The differences between scenarios diminished at higher restoration levels, likely due to the inclusion of randomly selected fields in all 30% restoration scenarios. Interestingly, pollination services showed a slight decline at the 3.3% restoration level (Fig. 3). See Fig. S8 in the Supplementary information for detailed ES distributions.

#### Impact of restoring randomly selected fields on ES supply

**Fig. 4:**
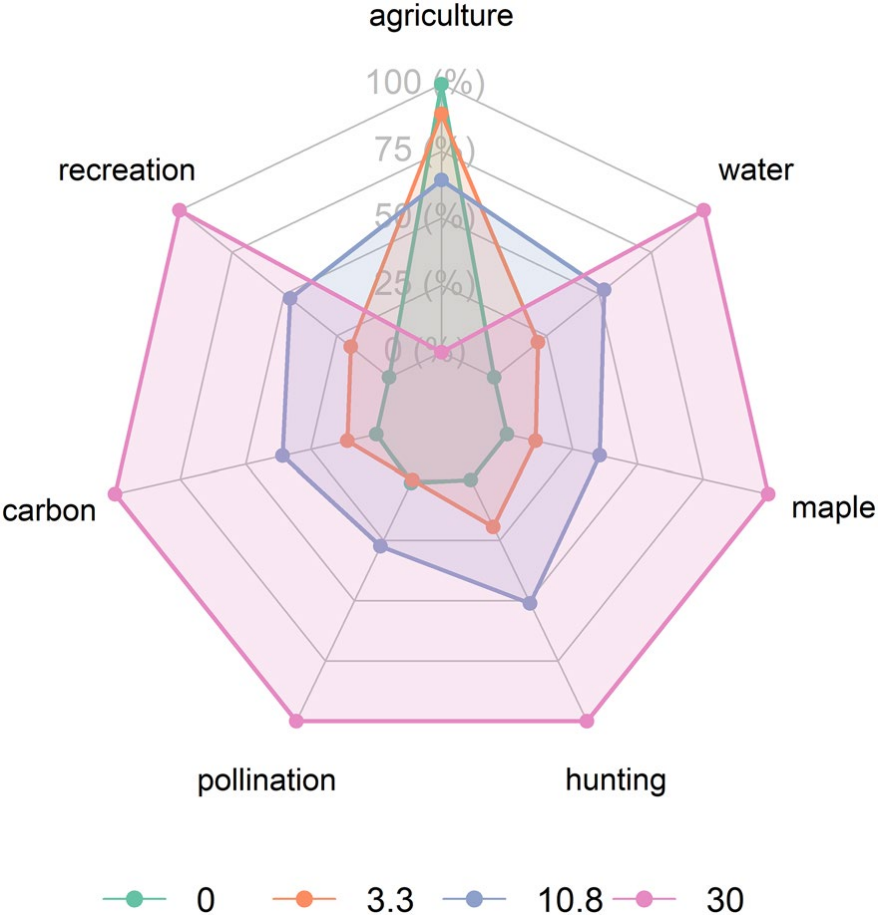
Radar chart illustrating the normalized sum of ES supply when restoring random fields under four scenarios: 0% (green), 3.3% (orange), 10.8% (purple), and 30% (pink). Each axis represents a different ES, with the grey lines indicating 0%, 25%, 50%, 75%, and 100% of the maximum potential supply for each service across all scenarios. Points on the lines correspond to the normalized sum of each service under the respective scenario, allowing for comparison of ES supply and its increase with restoration

Given the relatively small differences in ES supply between restoring abandoned, degraded, or randomly selected lands, we chose to focus our analysis from this point forward on scenarios featuring randomly selected fields, for simplicity. Increasing the restoration of agricultural sites to forest enhances the supply of most ES, apart from agriculture (Fig. 3). As illustrated in Fig. 3, when agricultural land is restored, crop production potential decreases since these areas are no longer available for farming. In contrast, the restoration leads to an increase in forest ecosystems, which provide greater capacity for other ES compared to agricultural lands. However, some services, such as hunting, quickly approach their maximum potential supply at low restoration levels, while others, including pollination, carbon sequestration, and maple production, exhibited minimal changes between 0% and 3.3% restoration levels (Fig. 4). Maple syrup production was the most impacted by restoration, with an overall increase of 36.1% in supply when restoring randomly 30%, followed by deer hunting (25.5%) and water quality (24.3%) Fig. 3.

**Fig. 5:**
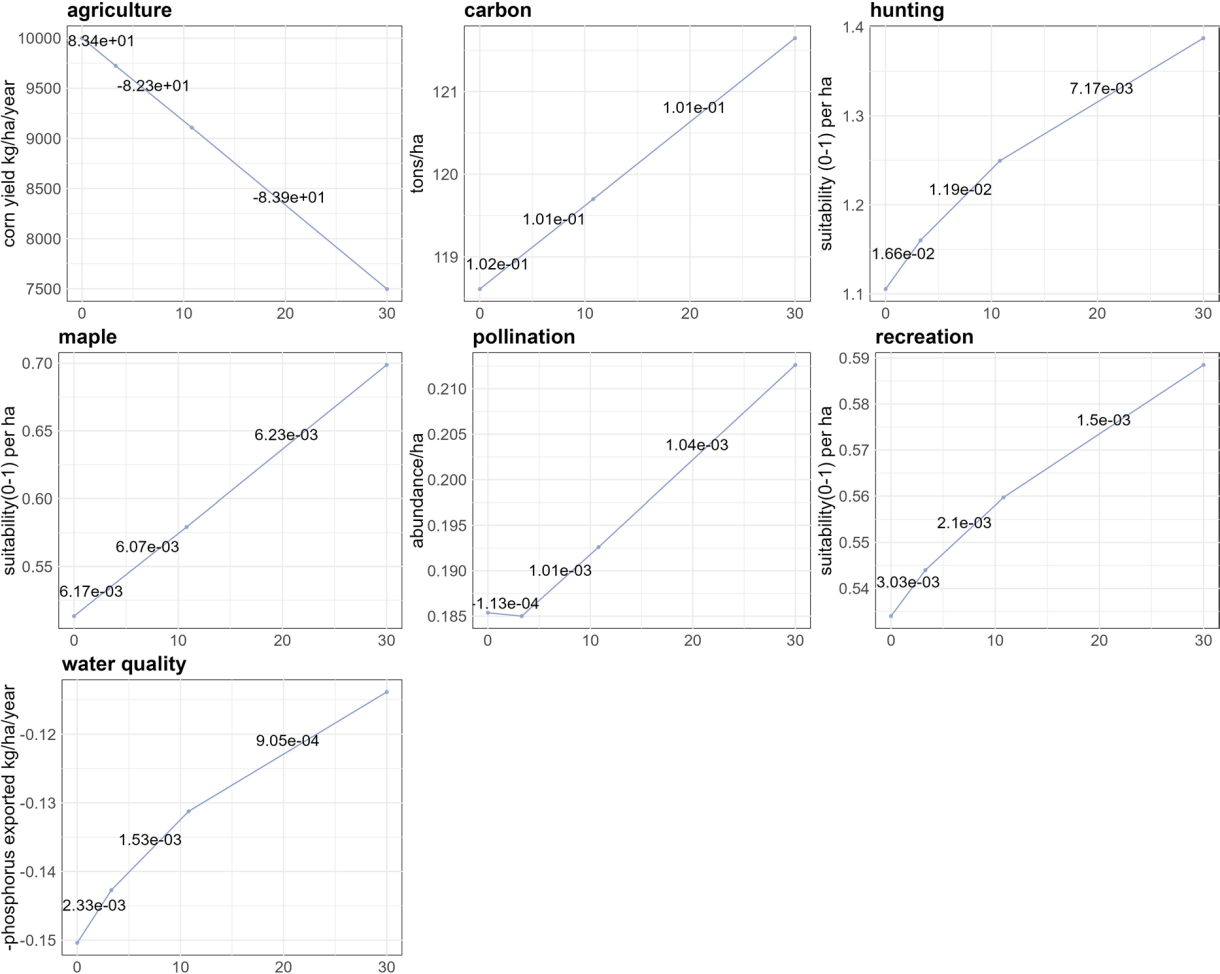
Each graph shows the cumulative supply of ecosystem service (ES) per hectare across the Montérégie region under random restoration scenarios, with varying percentages of field restoration. The y-axis represents the ES supply, while the x-axis indicates the percentage of restored area. Slope values along the lines indicate the change in ES supply per hectare per percentage restore over specific ranges: 0–3.3%, 3.3–10.8%, and 10.8–30%

Across scenarios, we observed a range of relationships between the area of restored random fields and ES supply. Although overall ES supply generally increased with amount of restored areas, agriculture exhibited negative supply trends with a decline of around 82 kg/ha/year per 1% increase in restored area. Pollination decreases solely when restoring between 0% and 3.3% of land (Fig. 4, Fig. 5). Beyond these directional trends, the relationships between ES supply and the extent of land restoration revealed variable rates of ES return per hectare. These rates followed two main patterns: linear and logarithmic. Services such as carbon, maple and agriculture exhibited linear trends, demonstrating a steady increase (or decrease in the case of agriculture) in supply as restoration progressed. Pollination displayed a linear increase in supply after its initial decline at 3.3%. Conversely, water quality, outdoor recreation, and hunting exhibited logarithmic trends, with a rapid initial increase in supply per hectare restored, that tapered off after 3.3% restoration as more land was restored (Fig. 5).

### Spatial patterns

#### Impact on landscape-scale ecosystem service supply

Across the landscape, the supply of most ES varies at the pixel level, with some zones benefiting from restoration (increased ES supply) and others not (decreased ES supply). However, the extent of these variations is influenced by the level of restoration, with some services showing more pronounced changes than others.

**Fig. 6:**
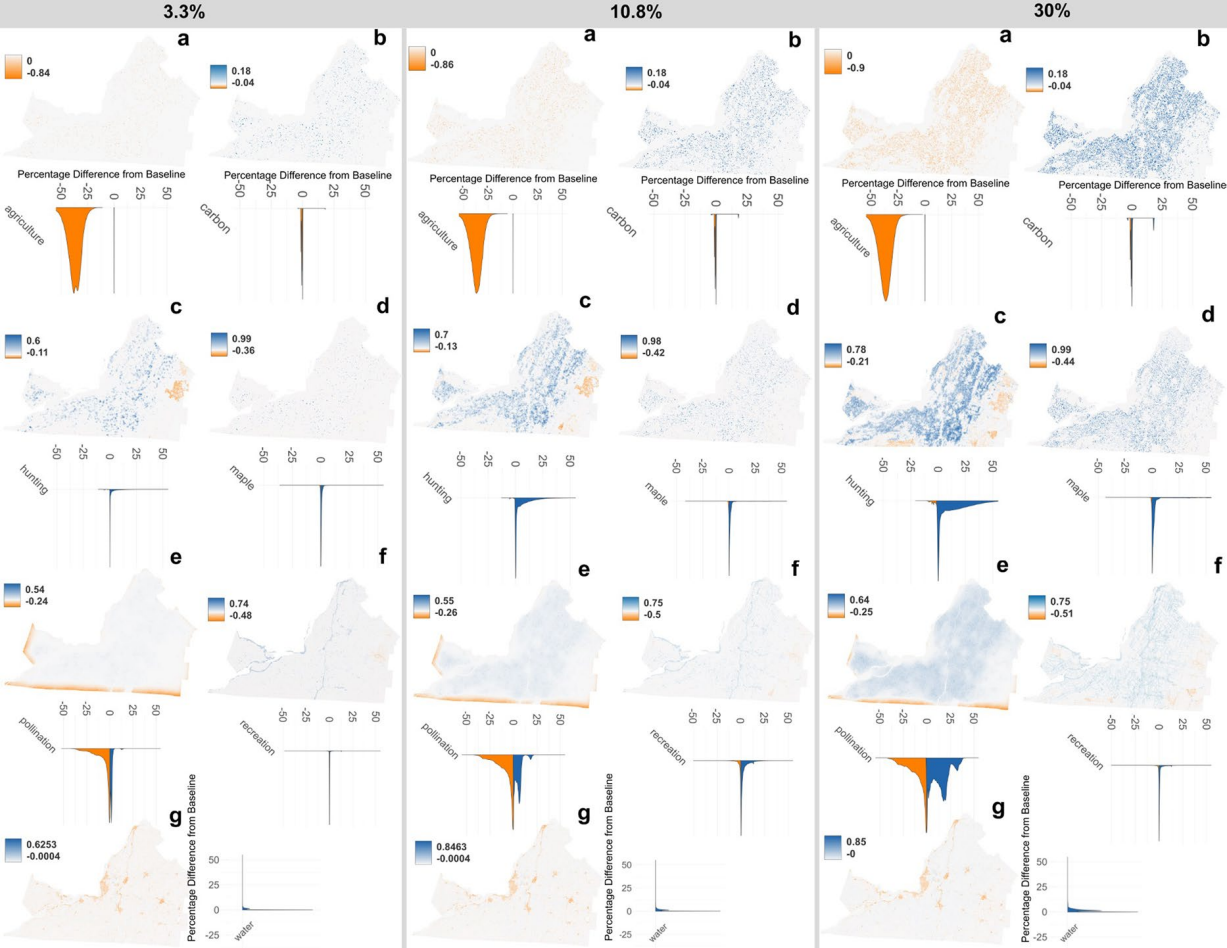
Map illustrating the location of change in ES supply under three restoration scenarios: 3.3%, 10.8%, and 30% random restoration. From left to right, each map represents the difference between the scenario and baseline at the pixel level for the following services: **a** Crop production, **b** Carbon storage, **c** White-tailed deer hunting, **d** Maple syrup production, **e**. Pollination, **f** Outdoor recreation and **g** Water quality. Each pixel’s value has been normalized to enable comparison across services. Below each map, a half-violin plot shows the distribution of pixel values for each service, illustrating how the supply changes compared to baseline. The orange color indicates a decline in supply, while blue represents an increase

**Restoring 3.3% of agricultural fields to forest** generated minor differences in ES supply for carbon, hunting, maple, outdoor recreation and water quality, which show a small increase in supply (Fig. 6. Panel 3.3% display b-d, f-g). However, agricultural yield declines in this scenario by around 38% compared to the baseline (Fig. 6. Panel 3.3% display a). Pollination supply displays mixed but generally minor changes across the landscape, with some zones experiencing an increase and others a decrease. The distribution of values is mostly concentrated around zero, indicating limited overall change (Fig. 6. Panel 3.3% display e). Overall, the changes for most ES are barely visible on the map, except for hunting (Fig. 6. Panel 3.3% display c) and pollination (Fig. 6. Panel 3.3% display e).

**Restoring 10.8% of agricultural fields to forest** generates more noticeable changes in ES supply compared to the 3.3% restoration scenarios. Hunting increases overall, but areas that were forested at baseline show a slight decrease, likely due to deer preference for edge habitats (Fig. 6. Panel 10.8% display c; see Fig. S11 for finer-scale maps). Maple syrup production increases in fragmented forest areas, but decreases in existing forest stands near restored fields, though the distribution of values clustered around zero, indicating minimal overall change (Fig. 6 Panel 10.8% display d). Pollination potential shows a decrease in some areas at the landscape edge, offset by an increase within the interior, possibly due to edge effects (Fig. 6. Panel 10.8% display e). For agricultural yield a decline similarly to the 3.3% restoration scenario is observed (Fig. 6. Panel 10.8% display a). However, other ES display an increase in supply with the 10.8% restoration compared to baseline, but most values remain close to zero, reflecting minimal overall change (Fig. 6. Panel 10.8% display b-g; see Fig. S11 for finer-scale maps).

**Restoring 30% of agricultural fields to forest** generates more visible variation in ES supply compared to lower restoration scenarios (Fig. 6. Panel 30%). At the landscape scale, zones of increase and decrease in ES supply become evident. Agricultural yield continues to decline in this scenario (Fig. 6. Panel 30% display a). Carbon storage shows an overall increase, particularly in agricultural zones, with a greater number of pixels showing improvements of around 25% compared to the baseline (Fig. 6. Panel 30% display b.). Hunting suitability increases in agricultural areas but decreases in already forested regions (Fig. 6. Panel 30% display c.). Similarly, maple syrup production suitability increases in fragmented forest areas, with minimal change in highly forested areas (Fig. 6. Panel 30% display d). For pollinator abundance, a notable increase occurs in agricultural zones, while declines in areas of initially low abundance are less pronounced at 30% restoration compared to 3.3% (Fig. 6. Panel 30% display e). In the case of outdoor recreation, a general increase in suitability is present, although densely forested areas with limited infrastructure see a decrease, similar to lower restoration levels (Fig. 6. Panel 30% display f.). Finally, water quality shows a clear improvement, with most pixel values indicating positive change (Fig. 6. Panel 30% display g). Interestingly, water quality increase near restored sites in developed land (see Fig. S11 for finer scale maps). Overall, the 30% restoration scenario results in the highest increase in service supply across all ES except agriculture, although most of carbon, maple syrup and outdoor recreation pixel values remain close to zero, indicating minimal overall change (half-violin plots, Fig. 6. Panel 30%).

#### Impact on ecosystem services spillover

**Fig. 7:**
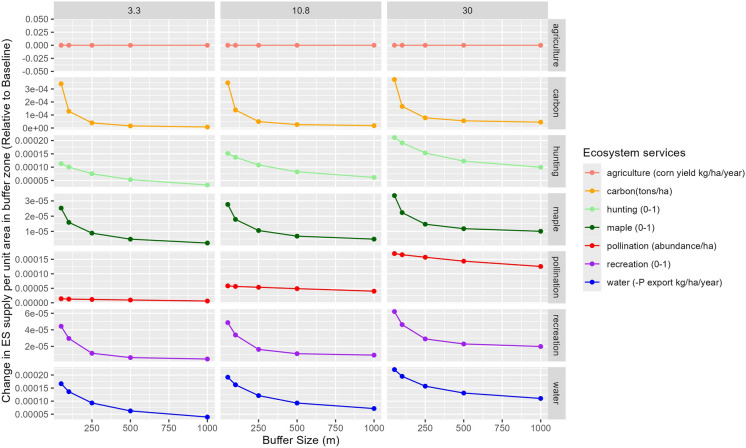
Spillover effect of ecosystem service (ES) supply, generated by restoring randomly 3.3%, 10.8%, and 30%. Each point displays the total variation in supply per unit area around the restored fields within a buffer zone of varying sizes (50, 100, 250, 500, 1000 m). The value displayed represents the difference from the baseline for each ES examined, excluding the direct ES values of the restored fields themselves

Comparing the baseline (0% restoration) ES supply to our restoration scenarios reveals spatial variability in supply both within and surrounding the restored sites (Fig. 7). In the random restoration scenarios, all services except agriculture show increased ES supply in areas surrounding the restored sites (Fig. 7; see Supplementary information, Fig S10-11, for a comparison across all scenarios). This variation is influenced by several factors, particularly buffer size. Most ES, excluding agriculture, experience a steady increase in supply up to 250–500 m from the restored site, after which the effect plateaus.

The extent of restoration also affects the intensity of the spillover. A higher percentage of restored land led to greater ES supply, especially at larger distances, for all services except agriculture (relative to the baseline). The spillover effect varies by ES type as well. While restoration does not impact agricultural supply in the surrounding areas, pollination shows only a small relative change within the 0–500 m buffer. In contrast, hunting, carbon sequestration, maple syrup production, outdoor recreation, and water quality services exhibit notable increases in supply up to 250–500 m from the restored sites in most scenarios (Fig. 7).

These spillovers are not always uniformly distributed around the restored sites. For example, in the case of outdoor recreation, we observe that highest spillover occurs on site edge close to the road, cities or other sites of recreation or transportation importance. Similarly, for hunting the highest spillover occurs in low forested areas and are more pronounced toward field edge, closest to the road (Fig. 6; see Figs. 16–17 for finer scale maps illustrating these observations.). For a detailed comparison of spillover effect between abandoned, degraded and random scenarios see Fig. S9 in the Supplementary information.

## Discussion

Our research shows that the extent and spatial distribution of restoration play a more important role in shaping the supply and distribution of ES than the initial condition of the land to be restored. There are three important outcomes worth further discussion. First, and perhaps unsurprisingly, the amount of land restored has the biggest impact on the ES outcomes. Second, the condition of the agricultural land being restored also plays an important role in outcomes. And finally, the spatial pattern of restoration has an important impact on ES outcomes, especially due to spillover effects, highlighting the importance of evaluating benefits both within and beyond the boundaries of restored sites for NbS such as forest restoration.

### Proportion of land restored and its impact on ES supply

Our scenarios indicate that increasing the proportion of agricultural land restored to forest enhances ES supply, except for agriculture yield, aligning with existing literature that highlights the greater capacity of forest ecosystems to provide multiple ES compared to agricultural land (Benayas et al. 2009; Newton et al. 2021; Hardaker et al. 2021; Lavorel et al. 2022). However, while the overall amount of restoration has a strong impact on ES supply, it does so at different rates for different services, following logarithmic, linear, or mixed trends. This suggests that while the extent of restoration is important, careful attention should be paid to the shape of the response curve for each service to optimize the amount of restoration relative to desired ES outcomes.

For hunting, outdoor recreation, and water quality, we observed a logarithmic increase in supply as restored area expanded, with a dramatic response to the initial restoration followed by less and less response per unit of area restored. For example, our water quality results show a sharp initial reduction in phosphorus pollution that tapered off with increasing restoration, consistent with previous findings (Scott et al. 2001; Lamb 2018). Similarly, hunting supply increased with initial restoration but plateaued as the restored forested area expanded, which could be explained by the clustering of restored fields reducing the availability of edge habitats preferred by deer (Alverson et al. 1988). This trend suggests that, for those ES with this logarithmic response to restoration, smaller restoration efforts can yield substantial initial benefits, with less impact of continued restoration. For these services, one might focus on smaller amounts of restoration to maintain agricultural production while enhancing other services at the same time.

In contrast, other ES, including carbon sequestration and maple syrup, had a more linear rate of increase with the amount of land restored (Cook-Patton et al. 2020; Paula et al. 2022). In these cases, continuing to restore further area can increase service provision. However, realized supply may be dependent on human intervention. For instance, maple syrup production is dependent on the human activity of tapping and boiling (Farrell 2013; Renard et al. 2016). Therefore, while our study shows a linear trend for the *potential* supply of maple syrup, realized production may show a different trend.

Yet other services had a mixed response to restoration. Pollination, for example showed a small decrease in supply between 0 and 3.3% restoration followed by a linear increase at higher levels of restoration. While this result contradicts some studies that have observed that small amounts of restoration increase pollination (Kremen and M’Gonigle 2015; Sexton and Emery 2020), it aligns with others suggesting that such interventions need to be densely distributed across a larger spatial scale, as simply increasing habitat does not guarantee improved pollination function (Breland et al. 2018; Donkersley et al. 2023). In our case, the initial decline in pollination might have been due to insufficiently dense restoration, resulting in forests that are too sparse to effectively support pollination at the landscape scale (Donkersley et al. 2023).

Overall, these results suggest that restoration of a given amount of agricultural area to forest will not have a similar effect on all services. The amount of land to be restored, the particular service in question, and the typical response curve of that service to the amount of land restored can be used to better estimate the response of each service to a given amount of restoration.

### The significance of the initial conditions of land restored in influencing ES supply

Our results also show that the initial conditions of the agricultural land restored plays a role in the outcomes for ES supply. While restoring random and degraded fields consistently reduces agricultural production, even at lower amounts of restoration, restoring abandoned fields presents a unique advantage of maintaining agricultural productivity while also improving provision of non-agricultural ES, at least at lower amounts of restoration (restoring 3.3% and 10.8% of the landscape in our study). However, this advantage disappeared when 30% of the land was restored; in this situation, the negative impacts on agricultural productivity were similar no matter the initial condition of the land restored. This likely occur because there was not enough abandoned land available to meet the higher restoration target, meaning more productive agricultural land had to be restored. This shift to restoring productive agricultural land leads to reduction in agricultural productivity. Therefore, the effects are highly dependent on the amount of abandoned land in the landscape. This aligns with previous studies, such as Torchio et al. (2024), who suggested achieving higher restoration targets may require restoring productive agricultural lands, leading to trade-offs in agricultural production.

While abandoned field restoration shows this distinct benefit for agricultural production at lower intensities, it generally yielded slightly lower ES supply in other categories compared to random or degraded field restoration. Although these differences were minor, they add to the literature by providing a landscape-scale comparison of the restoration of randomly selected, abandoned, and degraded fields—a comparison that, to our knowledge, has not been made in previous studies (Chazdon 2008; Benayas et al. 2009; Bullock et al. 2011; von Holle et al. 2020). The lower gains in abandoned fields for most ES may be linked to the specific conditions that led to their abandonment, such as poor soil quality or low productivity, which could limit ES supply in our scenarios (Alcantara et al. 2013; Yin et al. 2020). However, to fully understand the reasons behind the lower gains observed in abandoned fields in this study, future research could consider additional indicators, such as soil health or crop yield, rather than relying solely on theoretical yield derived from NDVI. Additionally, the spatial location of abandoned fields in the landscape may also be influencing their ES supply, as factors like accessibility or proximity to certain landscape element could play a role in the observed outcomes (Wu et al. 2022).

Here, our results suggest that if restoration objectives are low and a substantial amount of land is expected to be abandoned, prioritizing the restoration of abandoned fields rather than productive ones may be worthwhile, as it minimizes potential trade-offs with agricultural production. However, if restoration targets are high and the availability of abandoned land is limited, the effort required to identify and prioritize abandoned fields may not be justified, since, as shown by our results, a large proportion of the restored land will inevitably come from productive agricultural fields. In this context, the spatial distribution of restored fields within the landscape may be more important than their initial condition, since strategic placement can maximize the provision of other ES, such as water quality and erosion control, as illustrated in previous studies (e.g., Lamb et al. 2012; Barnett et al. 2016).

### The influence of restoration on spillover effect for enhancing ES supply

We found that there were two key elements which were pivotal for optimizing the ES benefits of restoration across the landscape: 1) the distribution of restoration sites and 2) the spillover effects of restoration beyond the restored site. Specifically, our results highlight how the surrounding landscape context influences hunting, maple syrup production, and suitability of the land for outdoor recreation. In our scenarios, we observed that areas with restoration sites near existing forests—especially those with infrastructure like roads nearby—tended to support higher outdoor recreation and hunting suitability. These observations align with previous studies noting deer preference for edge habitat (Williamson and Hirth 1985; Rieucau et al. 2007) and enhanced recreational use with higher accessibility, as road access for recreation is often located near edges (Spinney and Millward 2013). For water quality, we observed lower phosphorus pollution when restored sites were near poor nutrient retention areas like cities, which align with previous study finding (Clément et al. 2017). This suggests that practitioners should aim to clearly define restoration goals to target zones effectively, as for outdoor recreation and hunting services, fragmented restoration near forest patches with accessible edges appear to be beneficial, while improving water quality appear to require sites in areas prone to nutrient runoff. These results underscore the importance of tailoring NbS such as forest restoration to specific ecological features and service goals for greater effectiveness.

Beyond direct impacts, our analysis shows that restoration spillover effects extended ES supply up to around 500 m for all ES, except crop production, and up to one kilometer for some ES, such as water quality and hunting, with just 3.3% forest restoration. These findings are consistent with studies reporting benefits occurring at similar distances, including water quality improvements (Houlahan and Findlay 2004), pollination foraging distance (Redhead et al. 2016) and deer movement patterns for hunting services (Williamson and Hirth 1985; Rieucau et al. 2007). This suggests that recognizing the spatial extent of these spillover effects could allow managers to strategically place restoration sites at distances that maximize and compound these benefits, thereby optimizing the overall impact of NbS such as forest restoration at the landscape scale.

## Conclusion

Our findings suggest that targeting abandoned fields in agricultural landscapes can be a practical strategy for balancing ecological gains with minimal disruption to agricultural productivity, particularly at lower amounts of restoration. With restoration, it is important to consider potential trade-offs and the specific context of each landscape, such as the fact that strategies effective in agricultural areas may not be appropriate in urban settings. Our results suggest that the amount and spatial distribution of restored land are important elements for optimizing ES supply, while highlighting the potential influence of spillover effects in extending benefits beyond those sites that are restored. These findings underscore the value of implementing NbS such as forest restoration at strategic locations to enhance ES supply across landscapes. Overall, our results highlight the importance of identifying and prioritizing marginal lands within agricultural landscapes for potential restoration, with specific sites to be restored determined by a combination of overall area, location and connection to other forested or potential restoration sites, and the site condition. Planning restoration strategies from a landscape perspective can help achieve global and local policy goals. Future research should further explore the long-term impacts and potential trade-offs of restoration efforts to provide more comprehensive guidance for landscape planning.

## Supplementary Information

Below is the link to the electronic supplementary material.

Supplementary file1 (ZIP 14048 KB)

## Data Availability

All input data used in this study can be access from their original sources. All script used to run the analysis in this study can be accessed upon reasonable request from the corresponding author. Any supporting documentation can be accessed through the supplementary information joint to the manuscript.
