# Supplementary material for: Using old fields for new purposes: ecosystem service outcomes of restoring marginal agricultural land to forests
Source: Landsc Ecol. 2025 Jul 1;40(7):126. doi: 10.1007/s10980-025-02121-0 (PMC12213997; doi:10.1007/s10980-025-02121-0)
Supplement: Supplementary file 1 — Supplementary file1 (ZIP 14048 KB) [file 10980_2025_2121_MOESM1_ESM.zip › Supplementary_information_Destrempes/Supplementary_document_review.pdf]

Article title: Using old fields for new purposes: Ecosystem service outcomes of restoring marginal agricultural land to forests

Journal name: Landscape Ecology

Author names: Catherine Destrempes, Jesse T. Rieb, John Clark, Gabriela María Torchio, Brian Robinson, Monique Poulin and Elena M. Bennett.

Affiliation and e-mail address of the corresponding author: Catherine Destrempes; Department of Natural Resource Sciences, Faculty of Agricultural and Environmental Sciences, McGill University, 21111 Lakeshore Road, Ste. Anne de Bellevue, Québec, H9X 3V9, Canada; destrempescatherine@gmail.com

Supplementary information (SI)

The supplementary information section is divided into two categories: methodology and results. The methodology section offers additional explanations of the methods used in this study, along with tables and figures related to the dataset and variables. The results section provides a detailed analysis of model performance and includes extra data, figures, and information that support the main findings and analyses presented in the paper.

S1. Methodology supplementary information

1. Maxent modeling procedure

1.1. Introduction to Maxent

Maxent is a widely used software for species distribution modeling based on the maximum entropy theory (Li et al. 2020; Phillips et al. 2024). It uses presence points and environmental variables to generate probability distribution maps, indicating suitability from 0 (unsuitable) to 1 (highly suitable) (Fig. 3.9) (Merow et al. 2013; Phillips et al. 2017; Seda Arslan et al. 2021). Maxent's versatility extends beyond species distribution modeling to human activities and ES suitability distribution maps (Seda Arslan et al. 2021; Goodbody et al. 2021; Aouinti et al. 2022). While several customizable models like GLMs and GAMs could handle hunting, outdoor recreation, and maple syrup modeling, we settled on Maxent for its unique ability to work solely

with presence data. This decision stemmed from the challenge of obtaining reliable absence data, since lack of observation can not be counted as equivalent to absence (Elith et al. 2006).

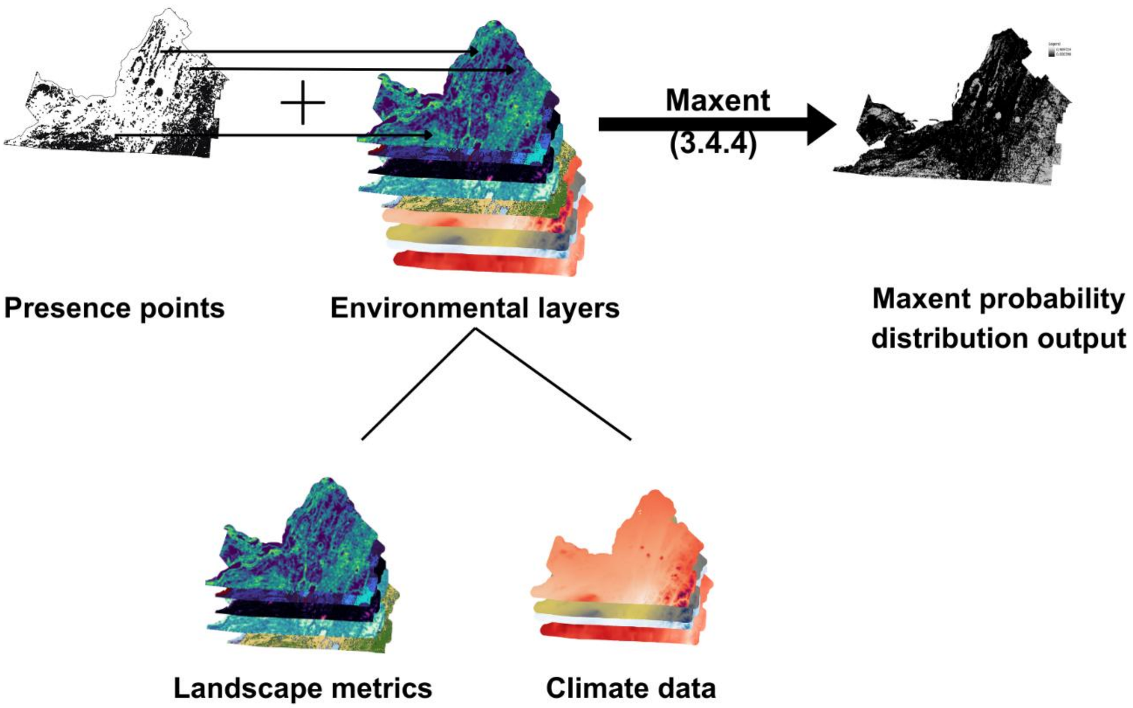

Fig. S1 Flow chart showing Maxent platform modeling process input and output inspired by Yan et al. (2018)

1.2. Environmental variables

In habitat suitability models like Maxent, it’s common to use predictor variables (referred to here as environmental layers) related to climate, vegetation, and topological characteristics of the environment that are hypothesized to influence the outcome of interest. In the case of the ecosystem services (ES), we suspected that landscape configuration and structure, which can be measured using landscape metrics, might be important predictors of the ES of interest (Ortner and Wallentin 2016). Landscape metrics are algorithms designed to describe and quantify these spatial patterns (Turner and Gardner 2015; Babí Almenar et al. 2018; Yaghoobi et al. 2022). We used multiple environmental raster layers grouped into climate data and landscape metrics (Fig. S1), integrating these variables to predict our ES of interest. All data was sourced from existing research, publicly available databases, and remote sensing tools.

1.2.1. Climate variables:

We sourced our climate data from the Worldclim database, which was highlighted as providing suitable variables for Maxent modeling in several previous studies (Fick and Hijmans 2017; Deb et al. 2020; Aouinti et al. 2022). I extracted all 19 variables from the Worldclim database at a 30 second resolution. Additional climate variables such as radiation, elevation, maximum and minimum temperature, were included due to their efficacy in modeling ES in the InVEST platform (Table S4) (Rieb and Bennett 2020).

1.2.2. Landscape metrics:

As our second type of environmental variable selected to predict ES delivery, we calculated various landscape metrics, considering the recognized importance of land use spatial patterns in determining ES delivery (Ziter et al. 2014; Mitchell et al. 2015; Rieb and Bennett 2020) (Fig. S1). We calculated nine metrics—contagion, patch size, Shannon diversity index, and six distances to edge measures, which indicates the proximity of a point to the nearest other kind of landscape, based on their significant contribution in predicting cultural services (Seda Arslan et al. 2021). To calculate and map each of these landscape metrics for all our scenarios we use diverse technique in RStudio (Posit team 2024).

I. Distance to edge:

To create distance-to-edge metrics, we generated binary raster maps representing the location of each edge element (e.g., forest, road) and measured the Euclidean distance for each pixel. We identified edge elements using land cover map (for forest and water), the OpenStreetMap (for recreation sites and trails) and the Canadian National Road Network (for road). These maps were then converted to binary rasters, where 1 represented the location of the edge element. Generating an R script with the *Raster* package *distance* function, we computed the Euclidean distances in meters from all NA pixels to the nearest non-NA pixel, creating maps that show the distance to each selected landscape element (recreation site, trail, road, water, or forest) for every

pixel in our study area. For distance to any edge, we adapted the script from Rieb and Bennett (2020), calculating Euclidean distances from the center of each pixel in the LULC map to the nearest pixel with a different LULC value within a 1 km radius.

II. Land use land cover:

To create the LULC maps for each scenario, we converted fields restored in any given scenario to forest values, using the Rieb & Bennett (2020) LULC map for 2014 as a baseline. This process generated LULC maps for each of our nine restoration scenarios.

III. Patch size:

To create the patch size map, we used the *landscapemetrics* package in R, we calculated patch sizes adhering to the 8-cell neighbourhood rule (McGarigal and Marks 1995; Hesselbarth et al. 2019; Rieb and Bennett 2020). We then used the *spatialize_lsm* function to associate each patch's size with its location, generating a raster map to use in Maxent (Hesselbarth et al. 2019).

IV. Shannon diversity index and contagion:

We use similar strategies to measure both contagion index and Shannon diversity index in this study. Shannon diversity index quantifies the diversity and evenness between elements such as land use and land cover categories in the landscape. Whereas contagion index, measures the probability of adjacent pixels belonging to different categories, indicating landscape habitat fragmentation (McGarigal and Marks 1995; Rieb and Bennett 2020). Both are normally measured for the entire landscape using the *landscapemetrics* package (v0.0); to obtain this measure for each individual pixel in our scenarios, we created a circular moving window of 1 km radius function in RStudio (Hesselbarth et al. 2019; Posit team 2024). This function extended the ability of the *landscapemetrics* package (v0.0) allowing us to measure both contagion and Shannon diversity at the pixel scale for each of our scenarios (the script is available upon request for further details) (Hesselbarth et al. 2019).

1.2.3. <u>Variable cleaning and processing:</u>

We processed each variable using a custom R script to ensure consistent extent, coordinate reference systems, resolutions, and handling of missing data (the script is available upon request for further details) (Yan et al. 2018).

1.3. Variable selection:

To determine which variables are useful predictors of hunting, maple syrup production, and outdoor recreation in our landscape, we used a stepwise approach to variable selection to determine the most contributive environmental layers for each ES model (Fig. 3.10). To do this, we extracted presence points for each ES and identified the environmental data that contributed most to predicting ES supply at these points in the Maxent platform (Fig 3.10).

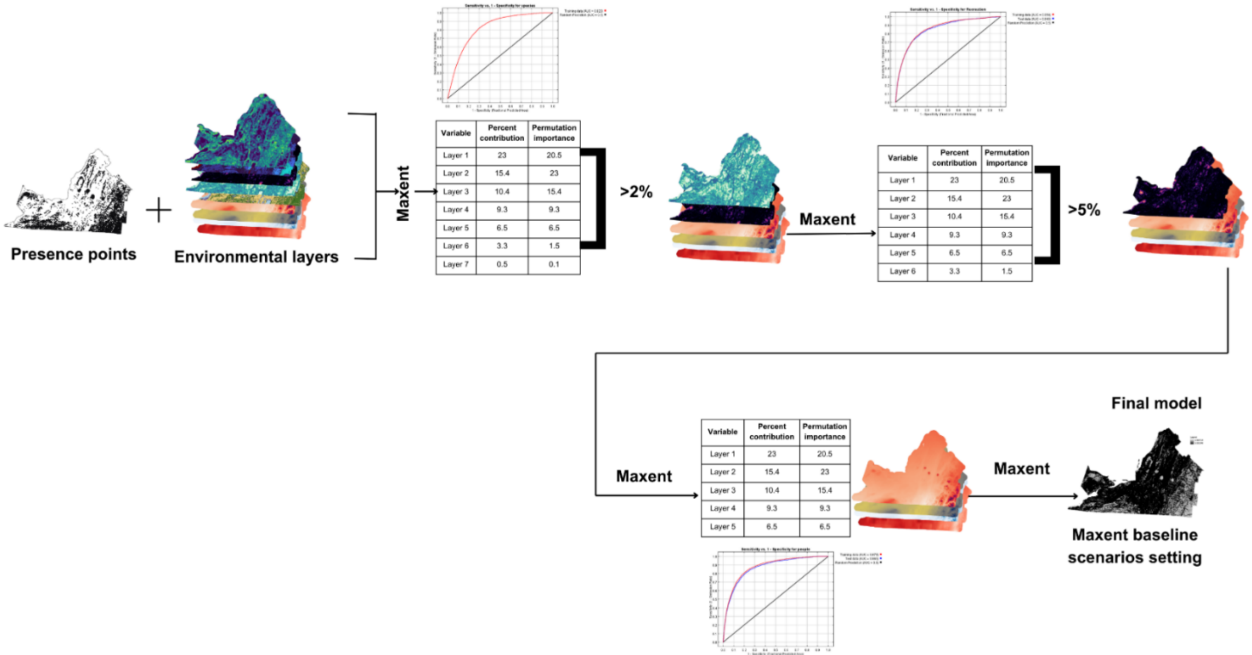

Fig. S2 Illustration of the stepwise variable selection process used to create the model for each ecosystem service (ES) evaluated using Maxent software. In this figure, “Maxent” represents the stages where the model is run in the software. The table displays the percentage contribution of each environmental variable to the model, and the graph shows the area under the curve (AUC), indicating model performance. Both values are generated by Maxent after each run and are used to identify variables to retain in the final model, thereby enhancing model performance and accuracy

1.3.1. Stepwise process:

To select the most suitable environmental layers to model our ES of interest in our scenarios, we began by generating a “base” model. This model incorporated presence points and all available environmental layers mentioned in section 1.2.2 (Fig. S2-3). Maxent uses background data points as pseudo-absences when generating the model (Phillips et al. 2024). To ensure randomness in selecting these background data points, we used Maxent’s built-in background selection feature to select them, maintaining a ratio of approximately 10 background points per occurrence point (Hysen et al. 2022). For replicability and to reduce computing time, we split our presence point dataset into two parts, allocating 70% for model training and using the remaining 30% for testing (Aouinti et al. 2022). This split helps prevent overfitting and evaluate the model’s predictive capability for our scenarios (Li et al., 2020; Merow et al., 2013).

Once the base model was generated, we evaluated the importance of each environmental layer. Maxent evaluates variable importance in the model through percent contribution and permutation importance metrics. Through regularization, these metrics are generated has a table after each run, ranking the variables based on their importance in predicting presence points (Phillips et al. 2006; Merow et al. 2013). This metric offers insights into which variables drive model predictions but should be used cautiously, as different algorithms might yield different contributions (Phillips 2017). We complemented this analysis with a jackknife test to provide another estimate of variable importance. Using this table, we retain only variables contributing more than 2%, forming a subset from the original set (Li et al. 2020). To address autocorrelation, we created a correlation matrix from this subset. The matrix helps us identify highly correlated variable pairs with correlation coefficients exceeding |0.7| (Fig. S3) (Deb et al. 2020). From each pair, we retained the variable with the highest contribution and reran the model in Maxent with the refined set of uncorrelated variables.

To ensure model performance was not compromised by our selection of predictor variables, we examined the area under the curve (AUC) after each run (Elith et al. 2006; Hill et al. 2012). The

AUC measures the model's ability to distinguish between presence and random background points. Specifically, it indicates how well the model ranks presence locations higher than random background locations (Phillips et al. 2006; Merow et al. 2013). AUC ranges from zero (unsuitable) to one (perfect suitability); an AUC below 0.5 suggests a model no better than random, while an AUC above 0.75 indicates a high-suitability model (Pearce and Ferrier 2000; Deb et al. 2020; Seda Arslan et al. 2021).We repeated this process until the model contained the most informative environmental variables for the presence locations. By informative we imply that variables were only kept at this step if their exclusion significantly decreased the model's AUC, this process allowed us to avoid multicollinearity issues (Hill et al. 2012; Goodbody et al. 2021).

Finally, using the model with the highest AUC, we created suitability maps for each ES and for each restoration scenarios, by creating a batch run in Maxent which would use the created model with the variable of the scenarios we generated to project a map of the ES in these circumstances. Each pixel in these maps is comprised in a continuous scale ranging from zero (unsuitable) to one (highly suitable), effectively mapping the potential for each scenario.

2. InVEST modeling data

We collected data from various sources to generate ecosystem service models for each scenario. Below, we outline all the data used in the InVEST platform (see Table S1-5) as well as the sources for each dataset used in this project (see Table S6) to run the water yield and nutrient delivery ratio modules.

Table S1 Biophysical table of water production we created and used in the InVEST annual water yield model. Each value represents the average value found for each corresponding land use and land cover (LULC) classes

Lucode	Land Use/Land Cover (LULC)	Vegetated indication (1-0)	Maximum root depth of plants (mm)	Crop coefficient
1	agriculture	1	1145	0.644693
2	orchard/vineyard	1	1500	0.767629
3	developed	0	-1	0.3864
4	water	0	-1	1
5	disturbed	1	3900	0.780446
6	shrubland	1	2600	0.752477
7	forest	1	3900	0.799481
8	peatland	0	-1	1.1
9	wetland	0	-1	1.1
10	marsh	0	-1	1.1
11	bare ground	0	-1	0.5
-9999	NA	0	0	0

Table S2 Biophysical table of phosphorus delivery on the landscape we created and used in the InVEST nutrient delivery ratio model. Each value represents the average value found for each corresponding land use and land cover (LULC) classes

**Lucode**	**Land Use/Land Cover (LULC)**	**Phosphorus load (kg/(ha · year))**	**Maximum phosphorus retention**	**Distance maximum nutrient retention (m)**	**Unadjusted Phosphorus load**	**Adjusted Phosphorus load**
1	agriculture	3.367398	0.5	30	1.683699	3.367398
2	orchard/vineyard	0.18	0.5	30	0.09	0.18
3	developed	19.5	0.1	30	1.95	19.5
4	water	0	0.1	30	0	0
5	disturbed	0.04	0.5	30	0.02	0.04
6	shrubland	0.025	0.8	30	0.02	0.025
7	forest	0.025	0.8	30	0.02	0.025
8	peatland	0	0.8	30	0	0
9	wetland	0	0.8	30	0	0
10	marsh	0	0.8	30	0	0
-9999	NA	0	0	30	0	0

Table S3 Carbon pool table we created and used in the InVEST carbon model. Each value represents the average carbon storage for the corresponding land use and land cover (LULC) classes (in t/ha)

Lucode	Land Use/Land Cover (LULC)	Carbon Stored in Aboveground Biomass	Carbon Stored in Belowground Biomass	Carbon Stored in Soil	Carbon Stored in Dead Organic Matter
1	Cropland	16.1	16.1	417.9	0
2	Orchards and Vineyards	16.24	16.24	418.64	0
3	Developed	22.11	22.11	467.88	0
4	Water	26.97	26.97	439.89	0
5	Cuts and regeneration	29.42	29.42	434.29	0
6	Shrublands	33.62	33.62	428.71	0
7	Forest	45.34	45.34	463.32	0
8	Peatland	42.03	42.03	493.13	0
9	Wetland	39.38	39.38	495.44	0
10	Marsh	0	0	0	0
-9999	No Data	0	0	0	0

Table S4 Guild table used in the InVEST crop pollination model. Values are from Zhang et al. (2024)

Species	Nesting suitability cavity index	Nesting suitability ground index	Foraging activity spring index	Foraging activity summer index	alpha	Relative abundance
bumble bees	1	1	1	1	1500	1

Table S5 Biophysical table used in the InVEST Crop pollination model. The input parameters are taken from Zhang et al. (2024)

**Lucode (*l*)**	**description**	**Nesting cavity availability index**	**Nesting ground availability index**	**Floral resources spring index**	**Floral resources summer index**	**Lucode Zhang (2024)**
1	Cropland	0.18	0.27	0.3	0.4	120
2	Orchards and Vineyards	0.18	0.27	0.3	0.4	120
3	Developed	0.2	0.24	0.43	0.53	34
4	Water	0	0	0	0	20
5	Cuts and regeneration	0	0	0	0	NA
6	Shrublands	0.67	0.76	0.7	0.58	50
7	Forest	0.68	0.66	0.58	0.46	200
8	Peatland	0.25	0.14	0.52	0.53	85
9	Wetland	0.25	0.14	0.52	0.53	80
10	Marsh	0	0	0	0	NA
-9999	No Data	0	0	0	0	NA

Table S6 Data and variables used to model and measure different ecosystem service (ES) of interest in this study

ES	Technique	Presence data	Model Variables (abbreviation use)	Dataset or source name	Year
Crop production	Dataset	NA	NDVI-based map of theoretical crop yield	(Rieb and Bennett 2020)	2014
Above-ground carbon storage	InVEST: Carbon storage and sequestration	NA	Carbon Stored in Soil	(Sothe et al. 2022)	2015-2019
			Carbon Stored in Belowground Biomass	(Spawn et al. 2020)	2010
			Carbon Stored in Aboveground Biomass	(Spawn et al. 2020)	2010
			Land use land cover (LULC)	Examine Scenarios LULC map generate from this study or MELCCFP (2015) land use map produce for the MDDELCC	2014
Pollination	InVEST: Crop pollination	NA	Guild table	Source from Zhang et al. (2024) under review, data originate from Agriculture and Agri-Food Canada (2014)	NA
			Biophysical table	Source from Zhang et al. (2024) under review, data originate from Koh et al. (2016)	NA
			Land use land cover (LULC)	Examine Scenarios LULC map generate from this study or MELCCFP (2015) land use map produce for the MDDELCC	2014
Water quality regulation	InVEST: Water Yield model	NA	Evapotranspiration (ET0)	Lehner et al.(2008), v1.1 hydrologically conditioned DEM	2000
			Land use land cover (LULC)	Examine Scenarios LULC map generate from this study or MELCCFP (2015) land use map produce for the MDDELCC	2014
			Biophysical table	Source from Rieb & Bennett (2020)	2014
			Minimum temperature, maximum temperature, precipitation (MinTemp, MaxTemp, Precip)	Source from Rieb & Bennett (2020) originate from (Thornton et al. 2017)	2014

			Plant available water content (PAWC)	Source from Rieb & Bennett (2020) originate from (Soil Landscapes of Canada Working Group 2010; Institut de recherche et de développement en agroenvironnement (IRDA) 2022)	Variable
			Watersheds	Source from Rieb & Bennett (2020) originate from HydroBasins level 8 from Lehner & Grill (2013)	2013
			Subwatersheds	Source from Rieb & Bennett (2020) originate from HydroBasins level 11 from Lehner & Grill (2013)	2013
			Root restricting depth (RRD)	Maximum root depth values source from Rieb & Bennett (2020), originate from multiple sources	Multiple
			z-parameter	Source from Rieb & Bennett (2020)	2013
	InVEST: Nutrient Deliver Ratio	NA	Land use land cover (LULC)	Examine Scenarios LULC map generate from this study or MELCCFP (2015) land use map produce for the MDDELCC	NA
			K_c	Source from Rieb & Bennett (2020) originate from (Allen et al. 1998)	1998
			Elevation (DEM)	Source from Rieb & Bennett (2020) originate from Lehner et al.(2008), v1.1 hydrologically conditioned DEM	2000
			Phosphorus loading by LULC class	Source from Rieb & Bennett (2020) data	2006
			Phosphorus retention efficiency by LULC class	Source from Rieb & Bennett (2020) data originate from (Qiu and Turner 2013)	Multiple
White-tailed deer hunting	Maxent	Deer kill locations	NA	Source from Renard et al.(2015) originate from Gouvernement du Québec, Système d'information sur la grande faune	2008-2012
			Annual Maximum temperature 1-km grid (MaxTemp)	Daymet accessible from (Thornton et al. 2017)	2014
			Shannon diversity index (shdi)	Calculated in the present study	NA

			Patch size (patchsize)	Calculated in the present study	NA
			BIO13: Precipitation of Wettest Month (mm) (layer.13)	(Fick and Hijmans 2017)	NA
			Distance to road (dteRoad)	Calculated in the present study using data from (NRN Statistics Canada 2024)	NA
			Distance to water (dteWater)	Calculated in the present study using data from (OSMF OpenStreetMap Foundation 2024)	NA
			Distance to Forest (dteForest)	Calculated in the present study	NA
Maple syrup production	Maxent	locations sugar maple forest	NA	forest inventories conducted by the MRNF (2016)	2001-2018
			Distance to Forest (dteForest)	Calculated in the present study using data from analyse LULC scenarios	NA
			Land use land cover (LULC)	Examine Scenarios LULC map generate from this study or MELCCFP (2015) land use map produce for the MDDELCC	2014
			(BIO6) Minimum Temperature of the Coldest Month (°C) (MinTemp)	(Fick and Hijmans 2017)	NA
			Patch size (patchsize)	Calculated in the present study using data from analyse LULC scenarios	NA
Outdoor recreation	Maxent	Geotagged photos	NA	Photos posted publicly on Flickr, including location, user, date and time, and a link to the photo	2000-2015
			Distance to recreation site (dteRecresite)	Calculated in the present study using data from (OSMF OpenStreetMap Foundation 2024)	NA
			Distance to any edge (dte)	Calculated in the present study using script originating from (Rieb and Bennett 2020)	NA
			Distance to trail (dteTrail)	Calculated in the present study using data from (OSMF OpenStreetMap Foundation 2024)	NA
			Distance to road (dteRoad)	Calculated in the present study using data from (NRN Statistics Canada 2024)	NA

	Distance to water (dteWater)	Calculated in the present study using data from (OSMF OpenStreetMap Foundation 2024)	NA
	Patch size (patchsize)	Calculated in the present study using data from analyse LULC scenarios	NA
	BIO13: Precipitation of Wettest Month (mm) (layer.13)	(Fick and Hijmans 2017)	NA
	Land use land cover (LULC)	Examine Scenarios LULC map generate from this study or MELCCFP (2015) land use map produce for the MDDELCC	2014

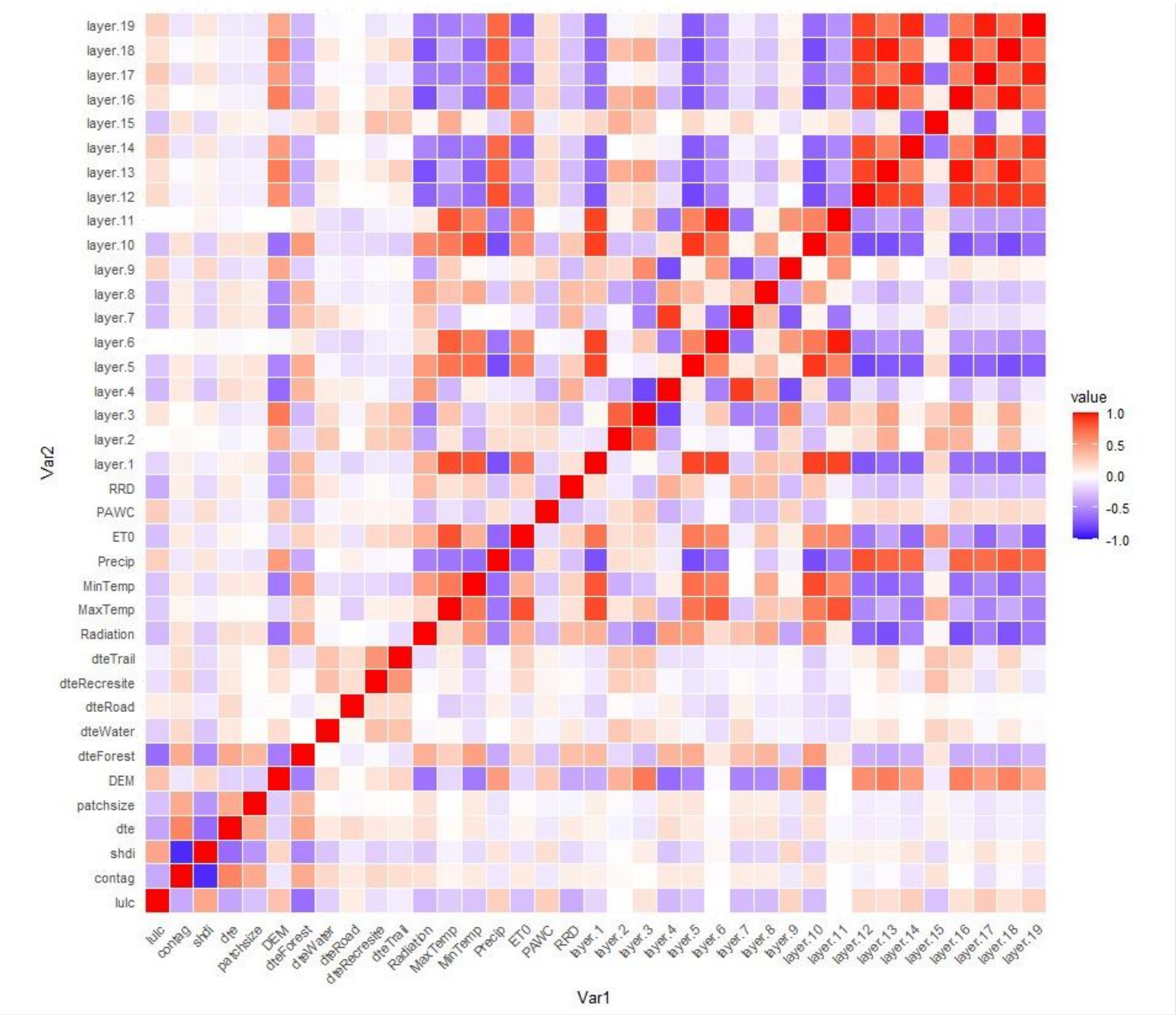

Fig. S3 Spearman's rank correlation coefficients for all variables included in the base model used for Maxent. The x-axis represents the first variable, while the y-axis represents the second variable being compared. Correlation values range from red (perfect positive correlation, 1) to white (no correlation, 0) to purple (perfect negative correlation, -1). Full variable names can be found in Table S6, except for the "layer" variable, which represents the 19 bioclimatic variables from Fick and Hijmans (2017)

3. Agriculture model data manipulation

To generate our agricultural map, we first created a baseline map using the NDVI-based crop production map (kg/ha/year) for 2014 developed by Rieb and Bennett (2020). While this map

included yield values for abandoned fields, these values reflected natural vegetation rather than crop production, as the fields were no longer under cultivation. To correct this, we assigned a value of 0 to all abandoned fields, identified through a polygon layer that we converted into a binary raster (0 for abandoned fields and 1 for others). By multiplying this binary raster with the Rieb and Bennett map, we excluded abandoned fields from the baseline agricultural yield calculations, creating a foundation for deriving maps for all subsequent scenarios.

Using our baseline map, we generated agricultural production maps for each of the nine restoration scenarios by creating a script that randomly selected fields for restoration from a polygon layer and converted it to a binary raster. Minor processing errors from the polygon-to-raster conversion caused negligible pixel misalignment, affecting 0.004% of the total agricultural land area. In these binary rasters, restored fields were assigned a value of 0, while all other fields retained a value of 1. Multiplying the baseline map with these rasters set crop production to 0 for restored fields while preserving original values for others, allowing us to generate maps of agricultural production in kg/ha/year for each scenario.

S.2. Results supplementary information

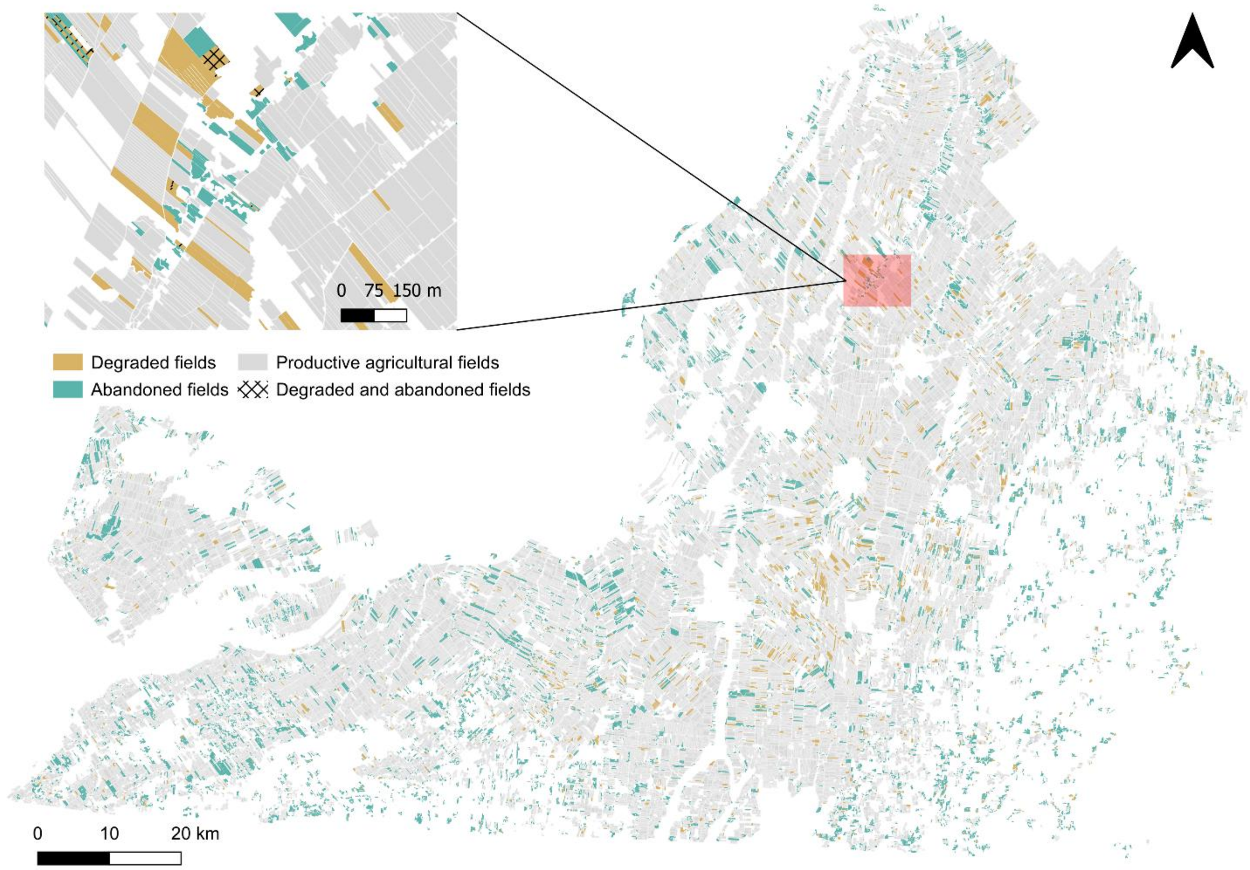

Fig. S4 Map showing the location of all fields identified as degraded (yellow), abandoned (blue), or productive (gray) in this study, presented for the entire Montérégie landscape (right) and a zoomed-in view (left). Fields classified as both abandoned and degraded are also indicated (black hatching)

1. Model performance and environmental variables

We generated three Maxent models to assess the suitability for outdoor recreation, white-tailed deer hunting, and maple syrup production across various scenarios. Detailed parameters and variables used in each model are summarized in Tables 6 and 7. The performance of each model is evaluated below.

1.1. Outdoor recreation

The Maxent model for outdoor recreation achieved an Area Under the Curve (AUC) of 0.854, indicating strong performance in distinguishing between presence and absence (Table S8; Fig. S6.a) (Pearce and Ferrier 2000). The model incorporated several variables, each contributing to varying degrees. Distance metrics related to recreation sites, trails, and roads collectively accounted for 67.7% of the model's suitability score (Table S8). Overall, landscape metrics contributed 91.3% to the model's predictive power, while climate variables accounted for only 8.6% (Fig. S5a-6a).

1.2. White-tailed deer hunting

The Maxent model for white-tailed deer hunting had an AUC of 0.767, suggesting moderate predictive capability (Table S8; Fig. S5.b) (Pearce and Ferrier 2000). Among the selected variables, distance to forest edge was the most influential, contributing 34% to the model's suitability score. This was followed by precipitation in the wettest month (Layer 13), contributing 23.7%. Landscape metrics accounted for 65.8% of the model's suitability score, while climate variables contributed 34.2% (Table S8; Fig. S5.b-6.b).

1.3. Maple Syrup Production

The Maxent model for maple syrup production exhibited the highest AUC of 0.876 among the three models, demonstrating strong performance in predicting suitable locations for sugar maple (Table S8). The most influential variable was distance to forest edge (dteForest), contributing 76.6% to the model's suitability score, followed by land use/land cover (lulc), which contributed 21%. This model's high reliance on these two variables suggests significant predictive potential, though the results should be interpreted with caution given the limited variable set. Landscape metrics accounted for 98.9% of the model's contribution, highlighting their critical role in predicting suitability (Table S8; Fig. S5.c-6.c). Our analysis indicates that the models perform well and can be relied upon to produce suitability maps for evaluating the potential of different scenarios. Across all three models developed using the Maxent platform, landscape metrics consistently had a greater influence on model predictions compared to climate variables.

Table S7 Number of sample points and model parameters used to project the potential distribution of outdoor recreation, hunting and maple syrup in each of the scenarios

**Ecosystem services**	**Presence point**		**Model parameter**	
	**Description**	**Number of points**	**Parameter**	**Value**
Outdoor recreation	Photograph location from flicker (weighted based # photographs/ user/days)	5870	Maximum background points	60000
			Regularization multiplier	1
			Random test percentage	30
			Feature type	Default
Deer hunting	Location where individual white tail deer were killed, 2008-2012	49726	Maximum background points	600000
			Regularization multiplier	1
			Random test percentage	30
			Feature type	Default
Maple syrup production	Forest inventorie conducted between 2001-2018	19425	Maximum background points	200000
			Regularization multiplier	1
			Random test percentage	30
			Feature type	Default

Table S8 Relevant predictor variables used to project the potential distribution of outdoor recreation, hunting and maple syrup in each of the nine scenarios. Visible are the relevant predictor variables used, and area under the curve (AUC) obtain when projecting the potential distribution of outdoor recreation, hunting and maple syrup in each of the nine scenarios

**Ecosystem services**	**Environmental variables**			
	**Variable included**	**Percent contribution**	**Permutation importance**	**AUC**
Outdoor recreation	layer13	0.8	3.9	0.854
	dteRecre	23	10.2	
	dte	3.3	1.7	
	dteTrail	29.3	20.5	
	dteRoad	15.4	33.8	
	dteWater	6.5	7	
	patchsize	9.3	10.9	
	lulc	10.4	11.1	
Deer hunting	layer 13	23.7	26.6	0.767
	layer 3	3.6	5.1	
	MaxTemp	7	3.8	
	shdi	16	18.9	
	patchsize	3.2	1.9	
	dteRoad	9.5	15.4	
	dteForest	34	20.3	
	dteWater	3.1	8	
Maple syrup production	layer 6	1.1	2.2	0.876
	patchsize	0.7	2.2	
	lulc	21.6	34.5	
	dteForest	76.6	59.8	

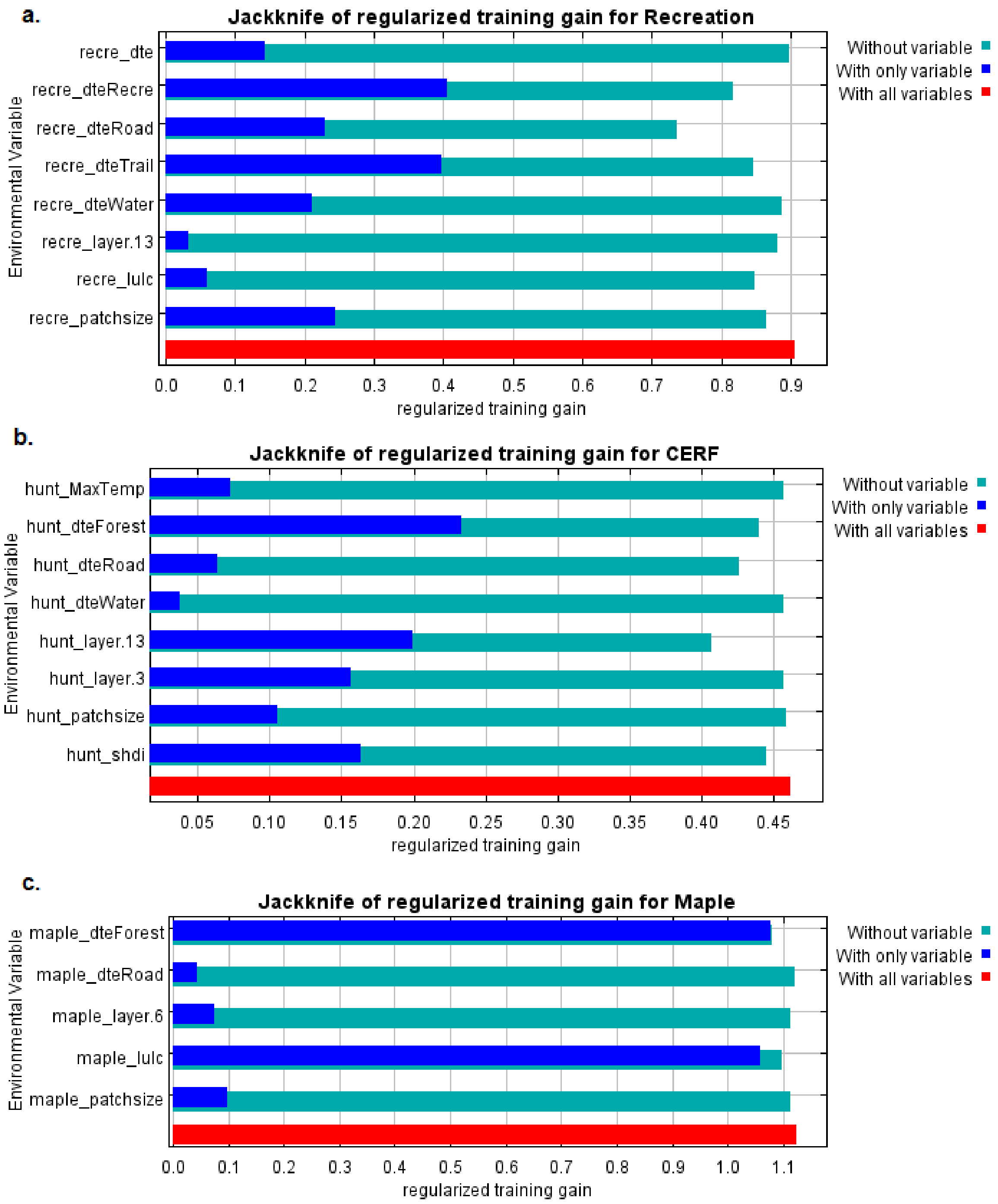

Fig. S5 Jackknife of regularize training gain for our three Maxent model; a) Outdoor recreation, b) White-tailed deer hunting and c) Maple syrup production. Variable importance was assessed by comparing the performance of models including only that variable (blue bar), excluding that variable (light blue bar), and including all variables (red bar)

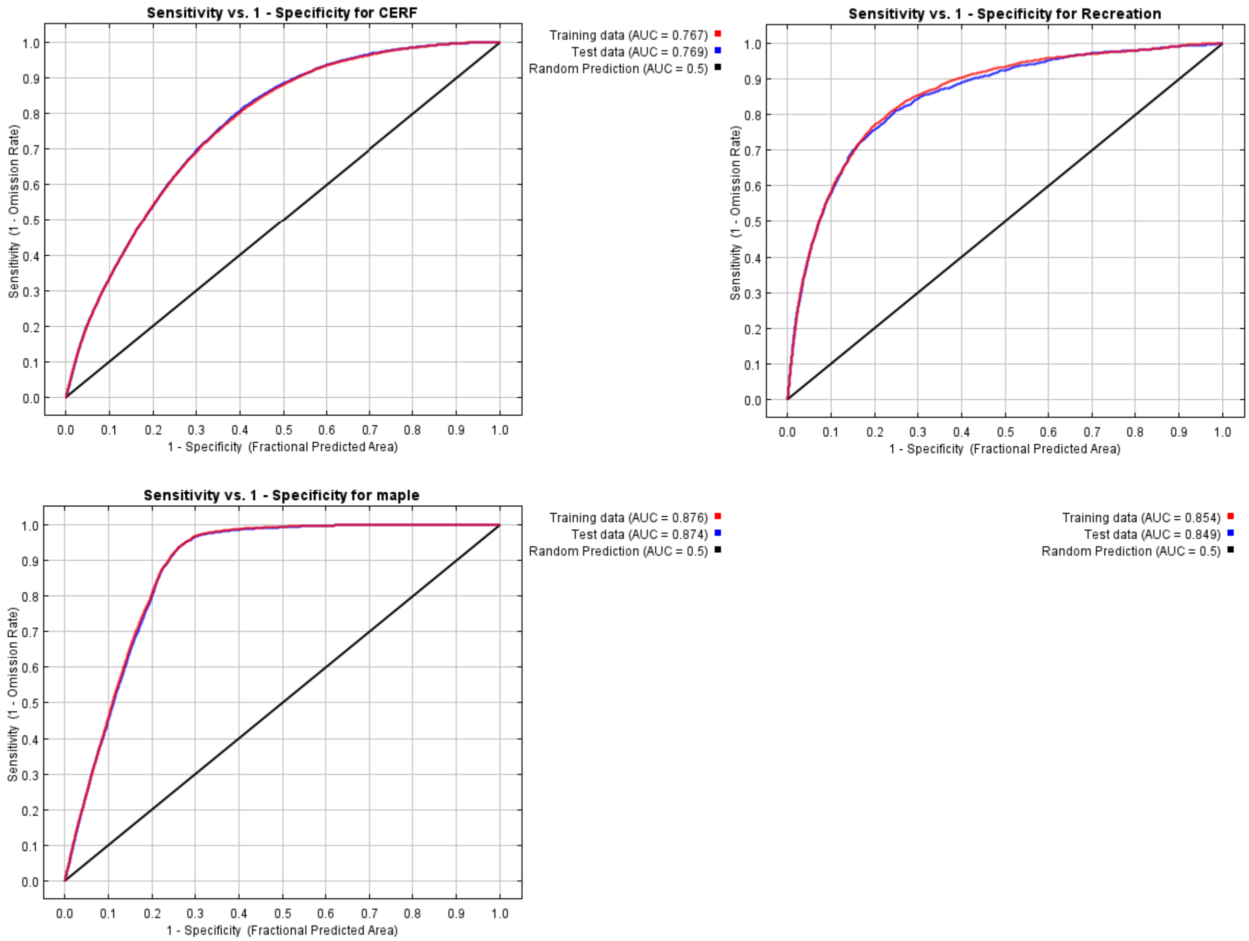

Fig. S6 The performance of each Maxent model was assessed by evaluating the area under the receiving operator curve (AUC): A) White-tailed deer hunting, B) Maple syrup production and C) Outdoor recreation

2. Interactions Among Ecosystem Services

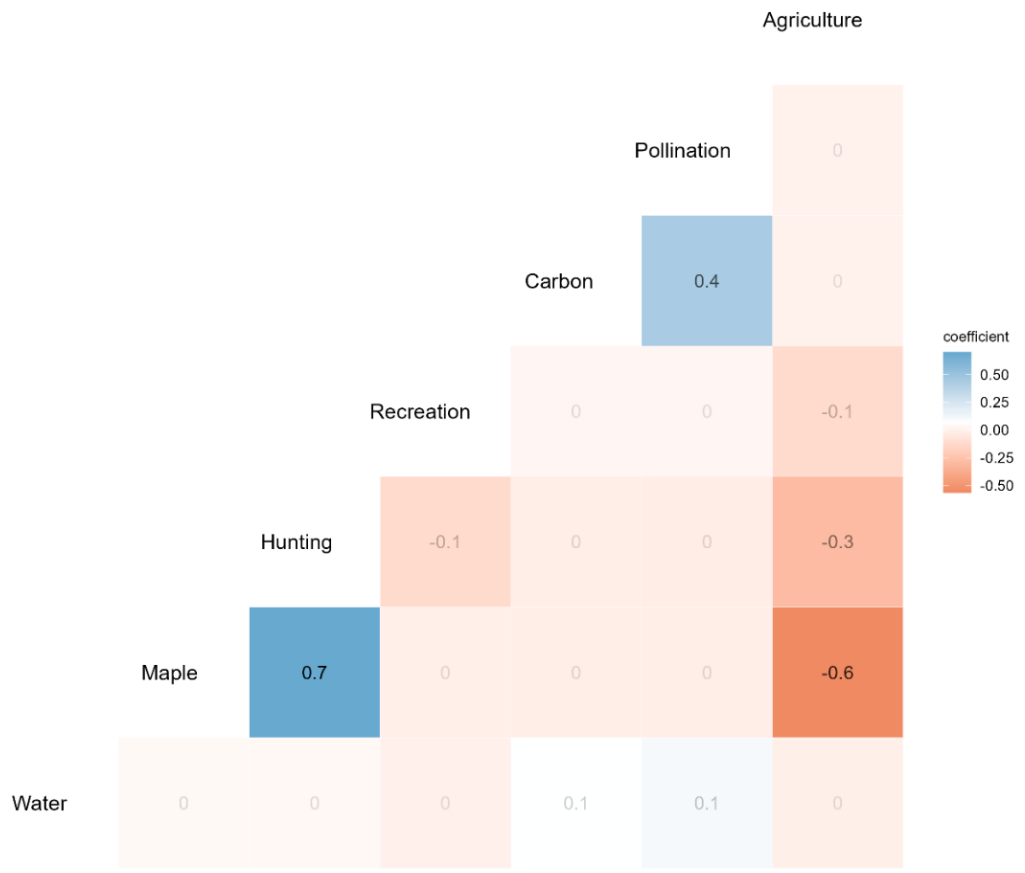

Fig. S7 Spearman pairwise correlation between ecosystem service (ES) at baseline conditions (2014). Orange signifies negative correlation and blue positive, with cell transparency representing the strength of the correlation between ES (strong correlation>=0.7 or -0.7)

3. Restoration scenarios complementary analysis and information

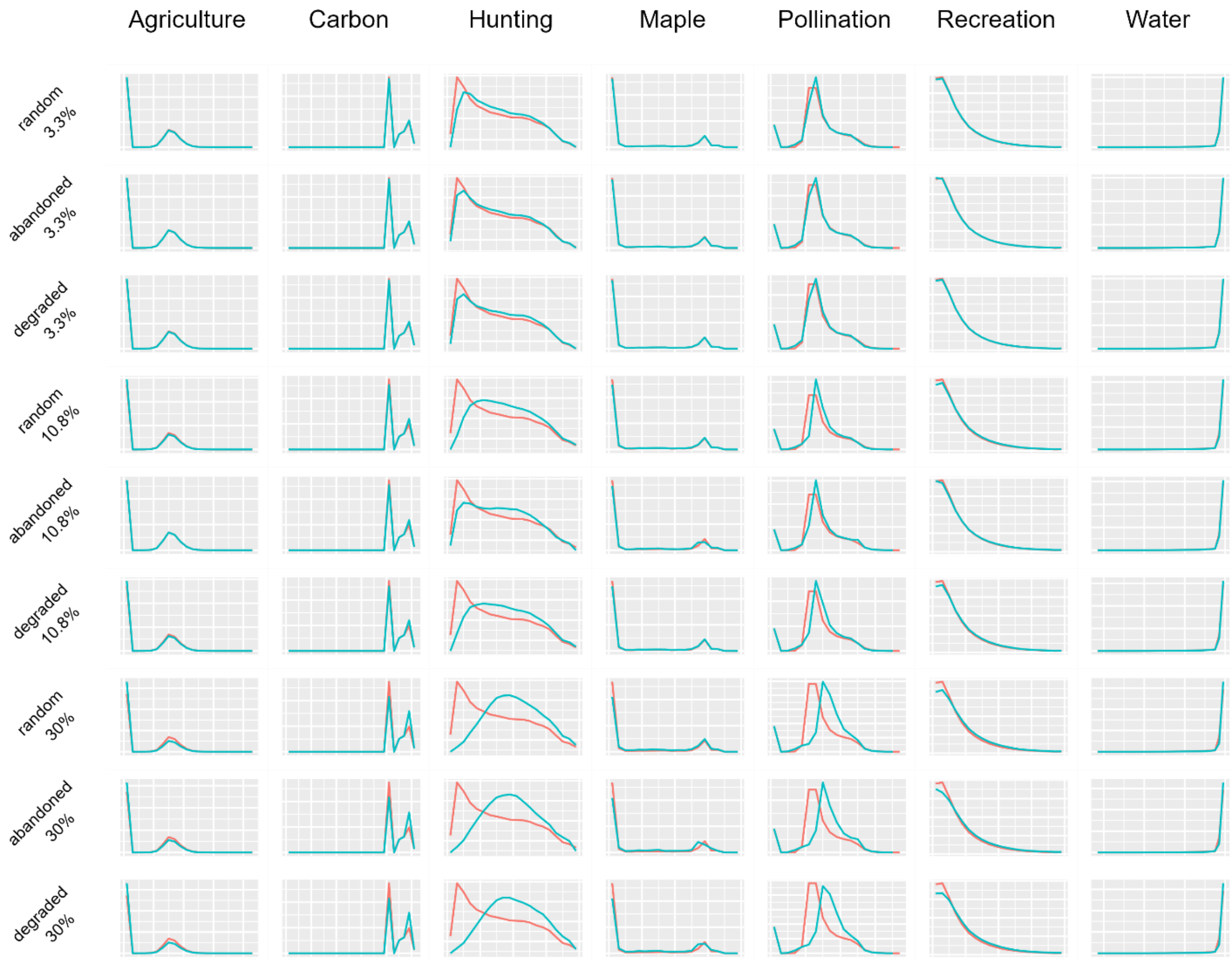

Fig. S8 Histograms illustrating the distribution of pixel values across rasters for each scenario in this study and their impact on the supply of seven ecosystem service (ES). Each histogram displays the frequency (y-axis) of specific pixel values (x-axis) for the rasters. Blue lines represent the pixel value distributions for the different scenarios, while red lines indicate the distribution for the baseline (0% restoration) scenario. This grouping helps to compare how different scenarios affect the supply of ES

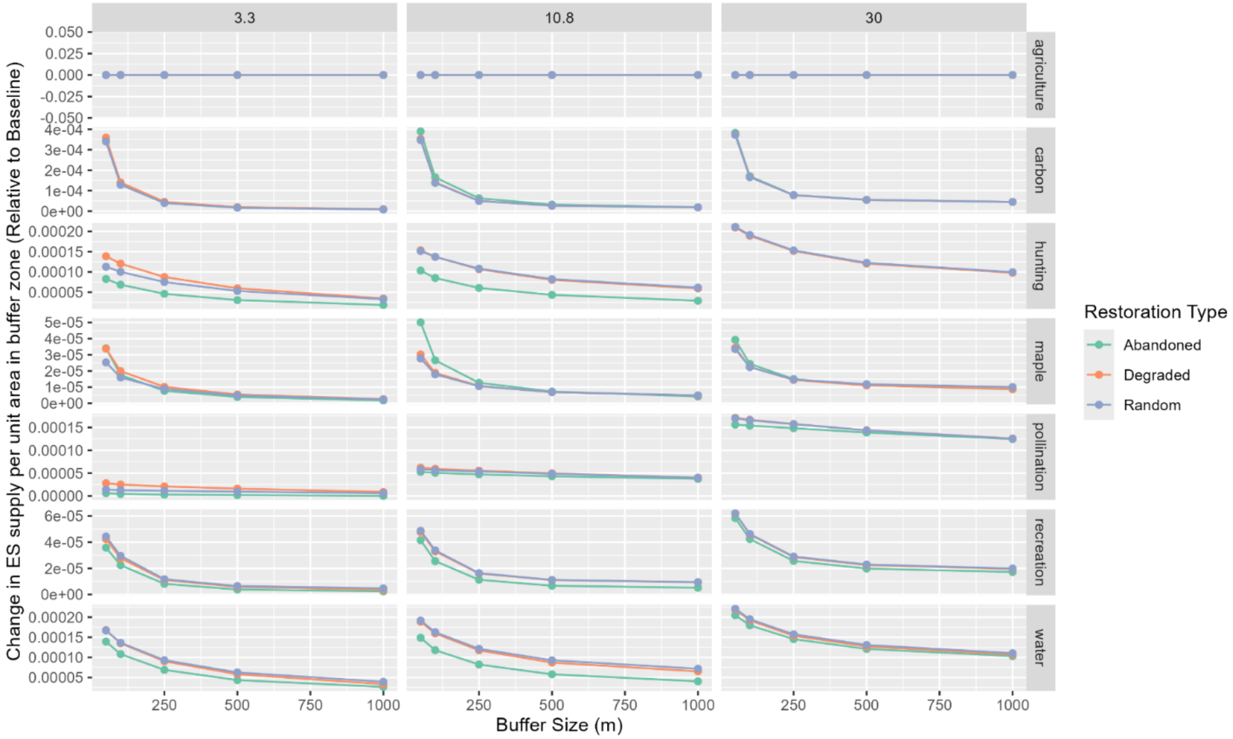

Fig. S9 Spillover effects of seven ecosystem services (ES) from restored sites in abandoned, degraded, and random scenarios at restoration levels of 3.3%, 10.8%, and 30%. The figure illustrates the variation in ES supply per unit area compared to the baseline (0% restoration) within buffer zones of different sizes (30, 50, 100, 250, 500, and 1000 meters) around the restored fields. Note that the direct ES values of the restored fields themselves are excluded. Each graph includes three lines representing the scenario types (abandoned, degraded, and random), distinguished by different colors for clarity. Each ecosystem service is presented with its own y-axis scale for clarity: agriculture (corn yield in kg/ha/year), carbon (tons/ha), hunting (suitability index 0–1), maple (suitability index 0–1), outdoor recreation (suitability index 0–1), pollination (abundance per ha), and water (phosphorus export in kg/ha/year)

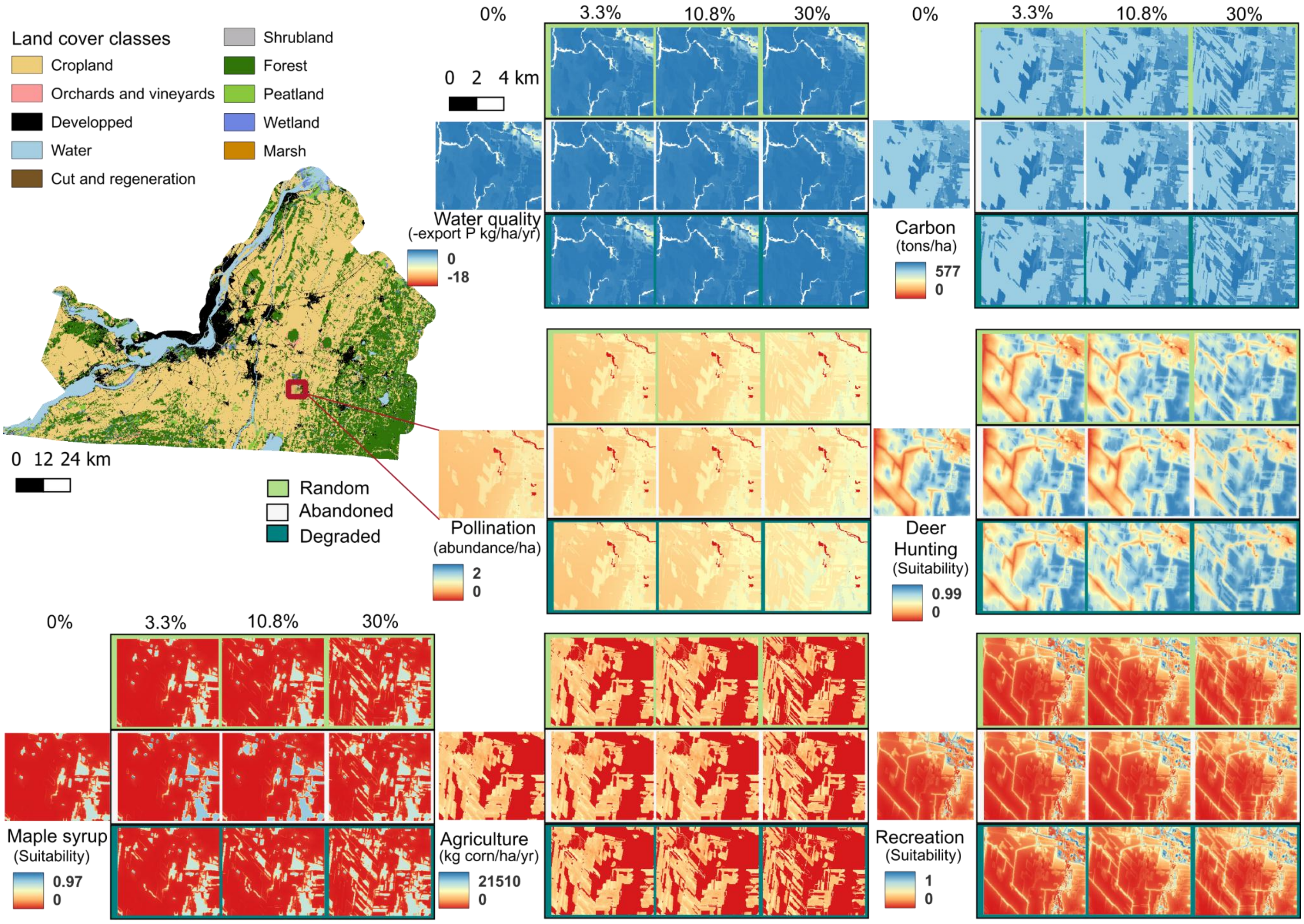
Land cover classes
Cropland
Orchards and vineyards
Developped
Water
Cut and regeneration
Shrubland
Forest
Peatland
Wetland
Marsh
0 12 24 km
Random
Abandoned
Degraded
0%
3.3%
10.8%
30%
0 2 4 km
Water quality
(-export P kg/ha/yr)
0
-18
Carbon
(tons/ha)
577
0
Pollination
(abundance/ha)
2
0
Deer
Hunting
(Suitability)
0.99
0
Maple syrup
(Suitability)
0.97
0
Agriculture
(kg corn/ha/yr)
21510
0
Recreation
(Suitability)
1
0

Fig. S10 Overview of all maps generated for this study. Each map displays one specific zone, highlighted by the red square on the Montérégie map in the upper left corner, for ease of visualization. Maps are organized by ecosystem service (ES) (noted on the left), restoration level (indicated above each box), and restoration type (colour-coded: green for randomly selected fields, grey for abandoned fields, and teal for degraded fields)

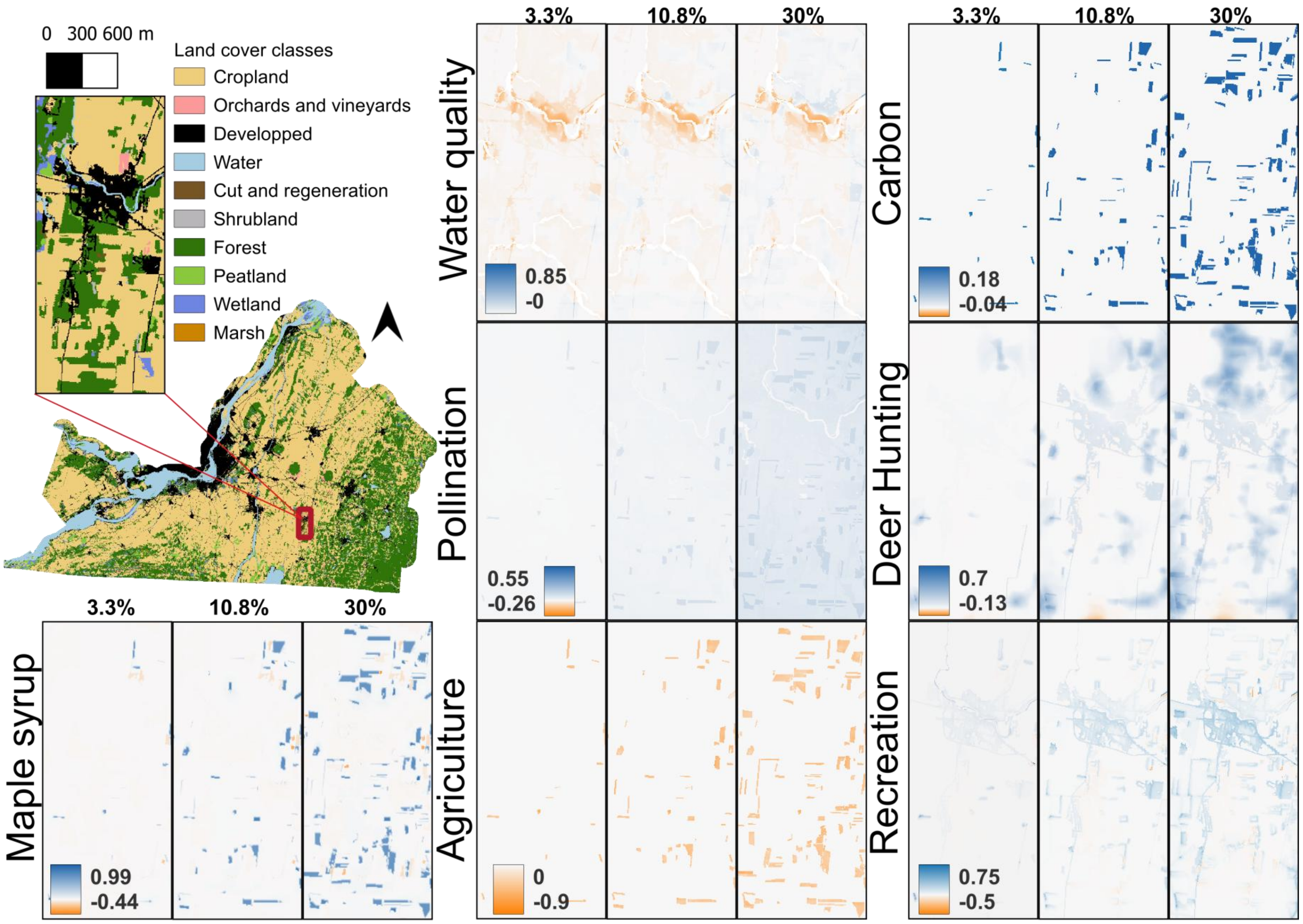
0 300 600 m
Land cover classes
Cropland
Orchards and vineyards
Developped
Water
Cut and regeneration
Shrubland
Forest
Peatland
Wetland
Marsh
3.3%
10.8%
30%
Water quality
0.85
-0
Carbon
0.18
-0.04
Pollination
0.55
-0.26
Deer Hunting
0.7
-0.13
Maple syrup
0.99
-0.44
Agriculture
0
-0.9
Recreation
0.75
-0.5

Fig. S11 Close-up view of the maps illustrating the normalized pixel increases (in blue) and decreases (in orange) from the baseline (0% restoration) in the supply of each ecosystem service (ES) under 3.3%, 10.8%, and 30% random restoration scenarios. The map in the left corner shows the landscape composition corresponding to the close-up views displayed for each ES under the respective restoration scenarios (3.3%, 10.8%, and 30%) in the random restoration maps

S.3. Supplementary sections references

Agriculture and Agri-Food Canada (2014) Native Pollinators and Agriculture in Canada. https://publications.gc.ca/collections/collection_2014/aac-aafc/A59-12-2014-eng.pdf. Accessed 2 Sep 2024

Allen R, Pereira L, Smith M (1998) Crop Evapotranspiration. Guidelines for Computing Crop Water Requirements. FAO

Aouinti H, Moutahir H, Touhami I, Bellot J, Khaldi A (2022) Observed and Predicted Geographic Distribution of *Acer monspessulanum* L. Using the Maxent Model in the Context of Climate Change. Forests 13:2049. https://doi.org/10.3390/f13122049

Babí Almenar J, Rugani B, Geneletti D, Brewer T (2018) Integration of ecosystem services into a conceptual spatial planning framework based on a landscape ecology perspective. Landsc Ecol 33:2047–2059. https://doi.org/10.1007/s10980-018-0727-8

Deb JC, Forbes G, MacLean DA (2020) Modeling the spatial distribution of selected North American woodland mammals under future climate scenarios. Mammal Rev 50:440–452. https://doi.org/10.1111/MAM.12210

Elith J, Graham C, Anderson RP, Dudík M, Ferrier S, Guisan A, Hijmans RJ, Huettmann F, Leathwick JR, Lehmann A, Li J, Lohmann LG, Loiselle BA, Manion G, Moritz C, Nakamura M, Nakazawa Y, Overton JMcCM, Townsend Peterson A, Phillips

SJ, Richardson K, Scachetti-Pereira R, Schapire RE, Soberón J, Williams S, Wisz MS, Zimmermann N (2006) Novel methods improve prediction of species' distributions from occurrence data. Ecography 29(2):129–151. https://doi.org/10.1111/j.2006.0906-7590.04596.x

Fick SE, Hijmans RJ (2017) WorldClim 2: new 1-km spatial resolution climate surfaces for global land areas. Int J Climatol 37(12):4302–4315. https://doi.org/10.1002/joc.5086. Accessed 1 Feb 2024

Goodbody TRH, Coops NC, Srivastava V, Parsons B, Kearney SP, Rickbeil GJM, Stenhouse GB (2021) Mapping recreation and tourism use across grizzly bear recovery areas using social network data and maximum entropy modeling. Ecol Model 440. https://doi.org/10.1016/j.ecolmodel.2020.109377

Hesselbarth MHK, Sciaini M, With KA, Wiegand K, Nowosad J (2019) landscapemetrics: an open-source R tool to calculate landscape metrics. Ecography 42(10):1648–1657. https://doi.org/10.1111/ecog.04617

Hill MP, Hoffmann AA, Mccoll SA, Umina PA (2012) Distribution of cryptic blue oat mite species in Australia: Current and future climate conditions. Agric For Entomol 14(2):127–137. https://doi.org/10.1111/j.1461-9563.2011.00544.x

Hysen L, Nayeri D, Cushman S, Wan HY (2022) Background sampling for multi-scale ensemble habitat selection modeling: Does the number of points matter? Ecol Inform 72:101914. https://doi.org/10.1016/j.ecoinf.2022.101914

Institut de recherche et de développement en agroenvironnement [IRDA] (2022) Carte pédologique du Québec. https://www.irda.qc.ca/fr/services/protection-ressources/sante-sols/information-sols/etudes-pedologiques/. Accessed 06 Mar 2024

Koh I, Lonsdorf EV, Williams NM, Brittain C, Isaacs R, Gibbs J, Ricketts TH (2016) Modeling the status, trends, and impacts of wild bee abundance in the United States. Proc Natl Acad Sci 113(1):140–145. https://doi.org/10.1073/pnas.1517685113

Lehner B, Grill G (2013) Global River Hydrography and Network Routing: Baseline Data and New Approaches to Study the World's Large River Systems. Hydrol Process 27:2171–2186. https://doi.org/10.1002/hyp.9740

Lehner B, Verdin K, Jarvis A (2008) New Global Hydrography Derived From Spaceborne Elevation Data. Eos Trans Am Geophys Union 89(10):93–94. https://doi.org/10.1029/2008EO100001

Li Y, Li M, Li C, Liu Z (2020) Optimized Maxent model predictions of climate change impacts on the suitable distribution of *Cunninghamia lanceolata* in China. Forests 11(3). https://doi.org/10.3390/f11030302

McGarigal K, Marks BJ (1995) FRAGSTATS: Spatial Pattern Analysis Program for Quantifying Landscape Structure. U.S. Department of Agriculture, Forest Service, Pacific Northwest Research Station, Portland, OR

Merow C, Smith MJ, Silander JA (2013) A practical guide to Maxent for modeling species' distributions: What it does, and why inputs and settings matter. Ecography 36(10):1058–1069. https://doi.org/10.1111/j.1600-0587.2013.07872.x

Ministère de l'Environnement, Lutte contre les changements climatiques, Faune et Parcs (2015) Utilisation du territoire. Données Québec. https://www.donneesquebec.ca/recherche/dataset/utilisation-du-territoire. Accessed 22 Jan 2021

Ministère des ressources naturelles et des forêts (2016) Carte écoforestière. Données Québec. https://www.donneesquebec.ca/recherche/dataset/carte-ecoforestiere-pdf. Accessed 06 Mar 2024

Mitchell MGE, Bennett EM, Gonzalez A (2015) Strong and nonlinear effects of fragmentation on ecosystem service provision at multiple scales. Environ Res Lett 10(9). https://doi.org/10.1088/1748-9326/10/9/094014

OpenStreetMap Foundation (2024). https://www.openstreetmap.org/copyright. Accessed 12 Mar 2024

Ortner OA, Wallentin G (2016) Estimating the benefit of landscape metrics in a Maxent model: Experimental application of landscape metrics surfaces at different scales. Master Thesis, University of Salzburg

Pearce J, Ferrier S (2000) Evaluating the predictive performance of habitat models developed using logistic regression. Ecol Model 133(3):225–245. https://doi.org/10.1016/S0304-3800(00)00322-7

Phillips SJ (2017) A Brief Tutorial on Maxent. American Museum of Natural History. https://biodiversityinformatics.amnh.org/open_source/Maxent/Maxent_tutorial_2021.pdf. Accessed 20 Mar 2024

Phillips SJ, Anderson RP, Dudík M, Schapire RE, Blair ME (2017) Opening the black box: an open-source release of Maxent. Ecography 40(7):887–893. https://doi.org/10.1111/ecog.03049

Phillips SJ, Anderson RP, Schapire RE (2006) Maximum entropy modeling of species geographic distributions. Ecol Model 190(3):231–259. https://doi.org/10.1016/j.ecolmodel.2005.03.026

Phillips SJ, Dudík M, Schapire RE (2024) Maxent software for modeling species niches and distributions. version 3.4.4. http://biodiversityinformatics.amnh.org/open_source/Maxent

Posit team (2024) RStudio: Integrated Development Environment for R. version 2024.4.2.764. https://www.rstudio.com/

Qiu J, Turner MG (2013) Spatial interactions among ecosystem services in an urbanizing agricultural watershed. Proc Natl Acad Sci U S A 110(29):12149–12154. https://doi.org/10.1073/pnas.1310539110

Renard D, Rhemtulla JM, Bennett EM (2015) Historical dynamics in ecosystem service bundles. Proc Natl Acad Sci 112(43):13411–13416. https://doi.org/10.1073/pnas.1502565112

Rieb JT, Bennett EM (2020) Landscape structure as a mediator of ecosystem service interactions. Landsc Ecol 35(12):2863–2880. https://doi.org/10.1007/s10980-020-01117-2

Seda Arslan E, Örücü ÖK (2021) Maxent modeling of the potential distribution areas of cultural ecosystem services using social media data and GIS. Environ Dev Sustain 23:2655–2667. https://doi.org/10.1007/s10668-020-00692-3

Soil Landscapes of Canada Working Group (2010) Soil Landscapes of Canada version 3.2. Agriculture and Agri-Food Canada. https://sis.agr.gc.ca/cansis/nsdb/slc/v3.2/index.html. Accessed 02 Sept 2024.

Sothe C, Gonsamo A, Arabian J, Kurz WA, Finkelstein SA, Snider J (2022) Large Soil Carbon Storage in Terrestrial Ecosystems of Canada. Glob Biogeochem Cycles 36(2). https://doi.org/10.1029/2021GB007213

Statistics Canada (2024) National Road Network - NRN - GeoBase Series - NRN Quebec SHAPE. https://open.canada.ca/data/en/dataset/3d282116-e556-400c-9306-ca1a3cada77f/resource/e945aef4-28ba-4900-926b-367cabc88339. Accessed 11 Mar 2024

Thornton MM, Thornton PE, Wei Y, Mayer BW, Cook RB, Vose RS (2017) Daymet: Annual Climate Summaries on a 1-km Grid for North America, Version 3. ORNL DAAC. https://doi.org/10.3334/ORNLDAAC/1343. Accessed 08 Aug 2024.

Turner MG, Gardner RH (2015) Landscape Ecology in Theory and Practice, Second Edition. Springer, New York

Yaghoobi M, Vafaeenejad A, Moradi H, Hashemi H (2022) Analysis of Landscape Composition and Configuration Based on LULC Change Modeling. Sustain Switz 14(20). https://doi.org/10.3390/su142013070

Yan Y, Kuo C-L, Feng C-C, Huang W, Fan H, Zipf A (2018) Coupling maximum entropy modeling with geotagged social media data to determine the geographic distribution of tourists. Int J Geogr Inf Sci 32(9):1699–1736. https://doi.org/10.1080/13658816.2018.1458989

Zhang Y, Thierry H, Cornejo L, Parrott L, Poulin M, Sherren K, Van Proosdij D, Robinson B (2024) Servicesheds connect people to the landscapes upon which they depend. People nat 00:1–15. https://doi.org/10.1002/pan3.10762

Ziter C, Bennett EM, Gonzalez A (2014) Temperate forest fragments maintain aboveground carbon stocks out to the forest edge despite changes in community composition. Oecologia 176(3):893–902. https://doi.org/10.1007/s00442-014-3061-0
