# Supplementary figures and images for: Using old fields for new purposes: ecosystem service outcomes of restoring marginal agricultural land to forests
Source: Landsc Ecol. 2025 Jul 1;40(7):126. doi: 10.1007/s10980-025-02121-0 (PMC12213997; doi:10.1007/s10980-025-02121-0)

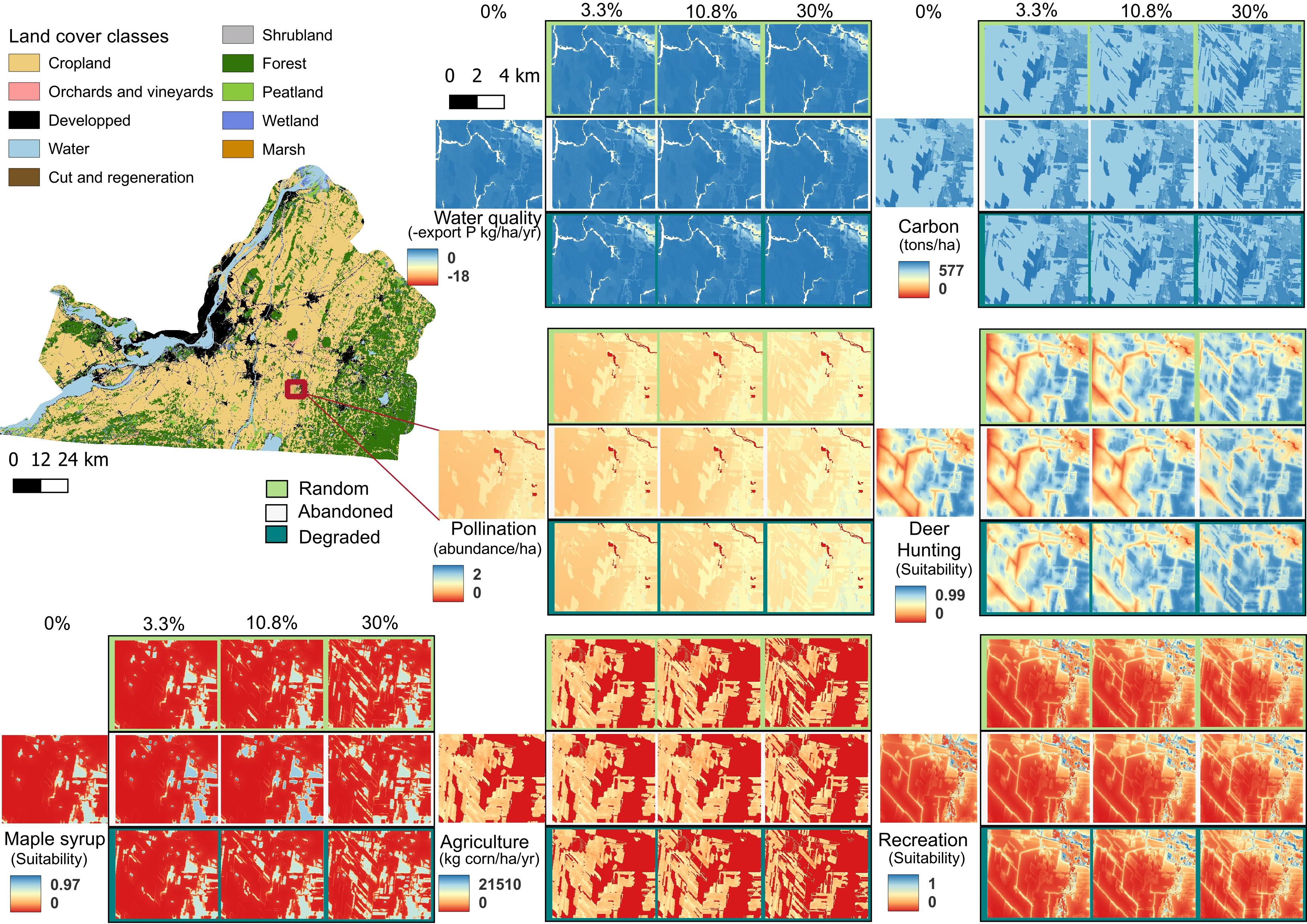

Supplement: Supplementary file 1 — Supplementary file1 (ZIP 14048 KB) [file 10980_2025_2121_MOESM1_ESM.zip › Supplementary_information_Destrempes/Image/Fig. S10.qgis.jpg]

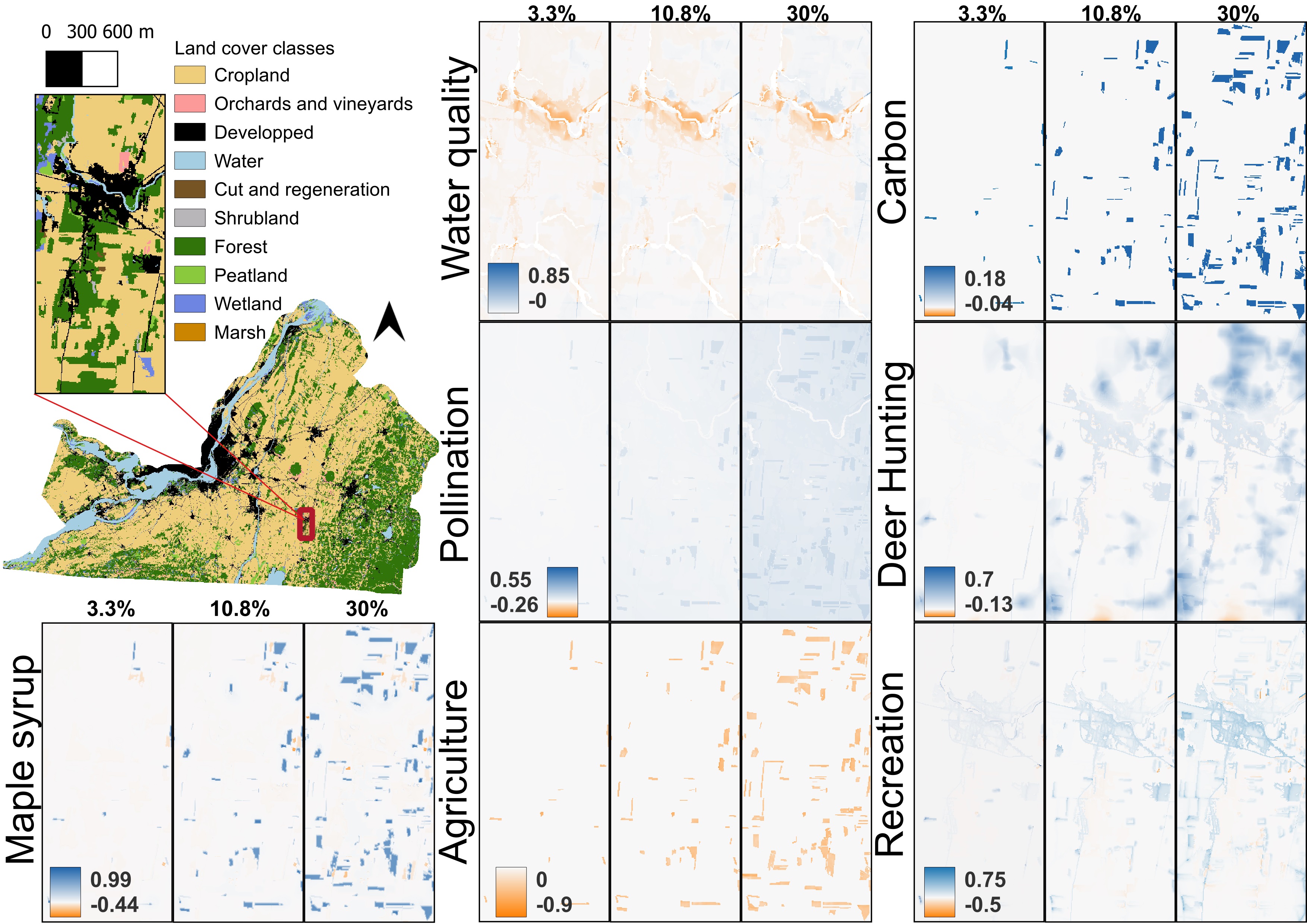

Supplement: Supplementary file 1 — Supplementary file1 (ZIP 14048 KB) [file 10980_2025_2121_MOESM1_ESM.zip › Supplementary_information_Destrempes/Image/Fig. S11.qgis.jpg]

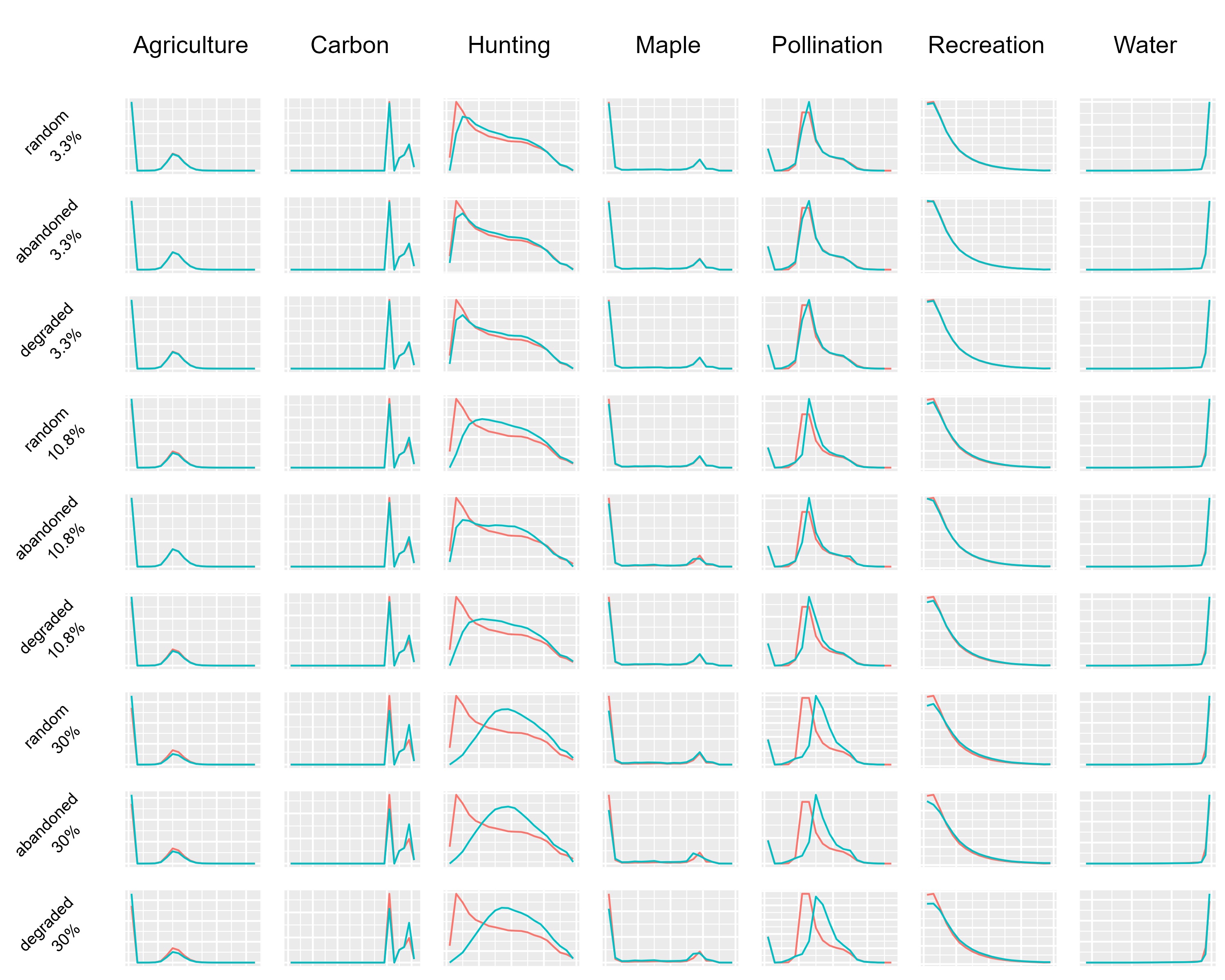

Supplement: Supplementary file 1 — Supplementary file1 (ZIP 14048 KB) [file 10980_2025_2121_MOESM1_ESM.zip › Supplementary_information_Destrempes/Image/Fig. S8_r.jpg]

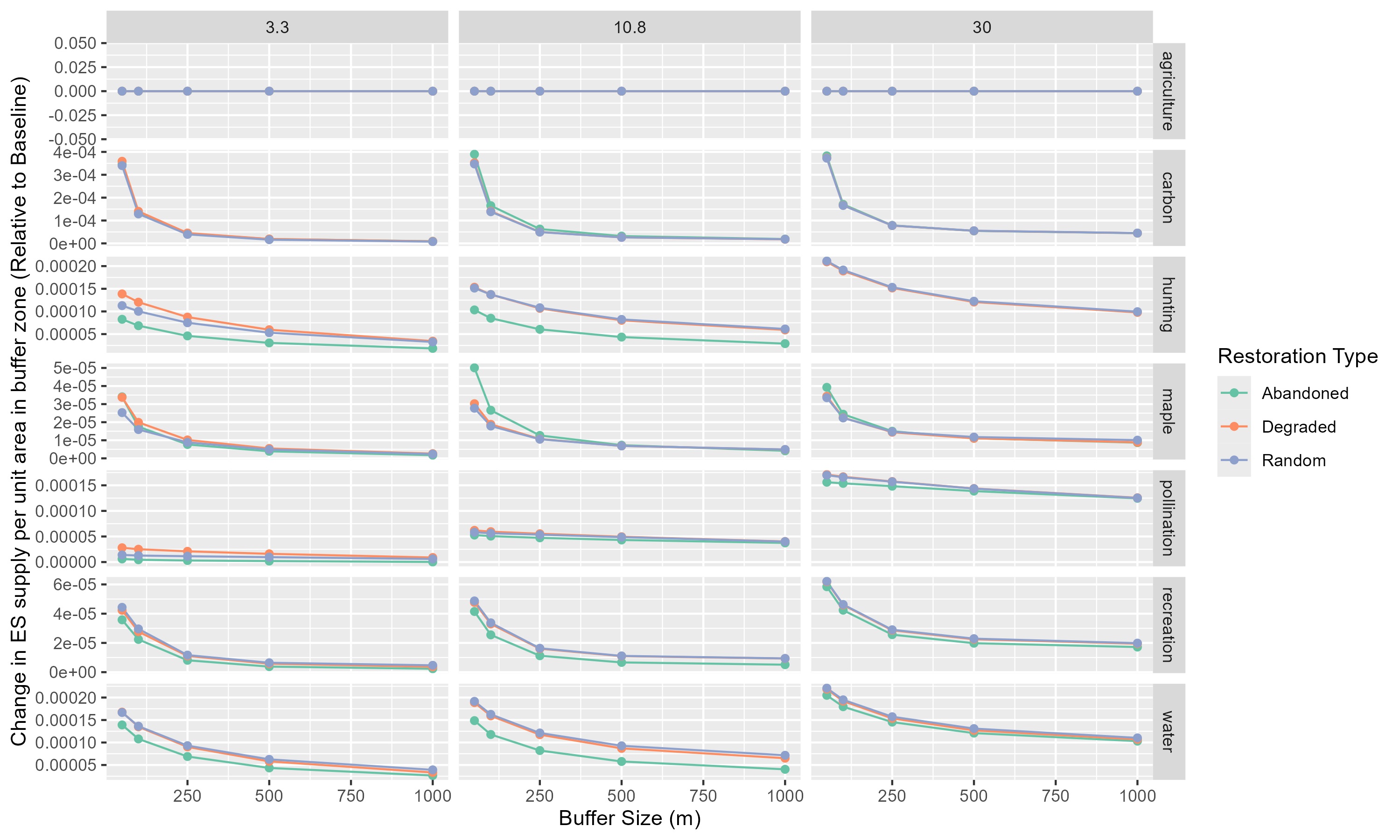

Supplement: Supplementary file 1 — Supplementary file1 (ZIP 14048 KB) [file 10980_2025_2121_MOESM1_ESM.zip › Supplementary_information_Destrempes/Image/Fig. S9_r.jpg]

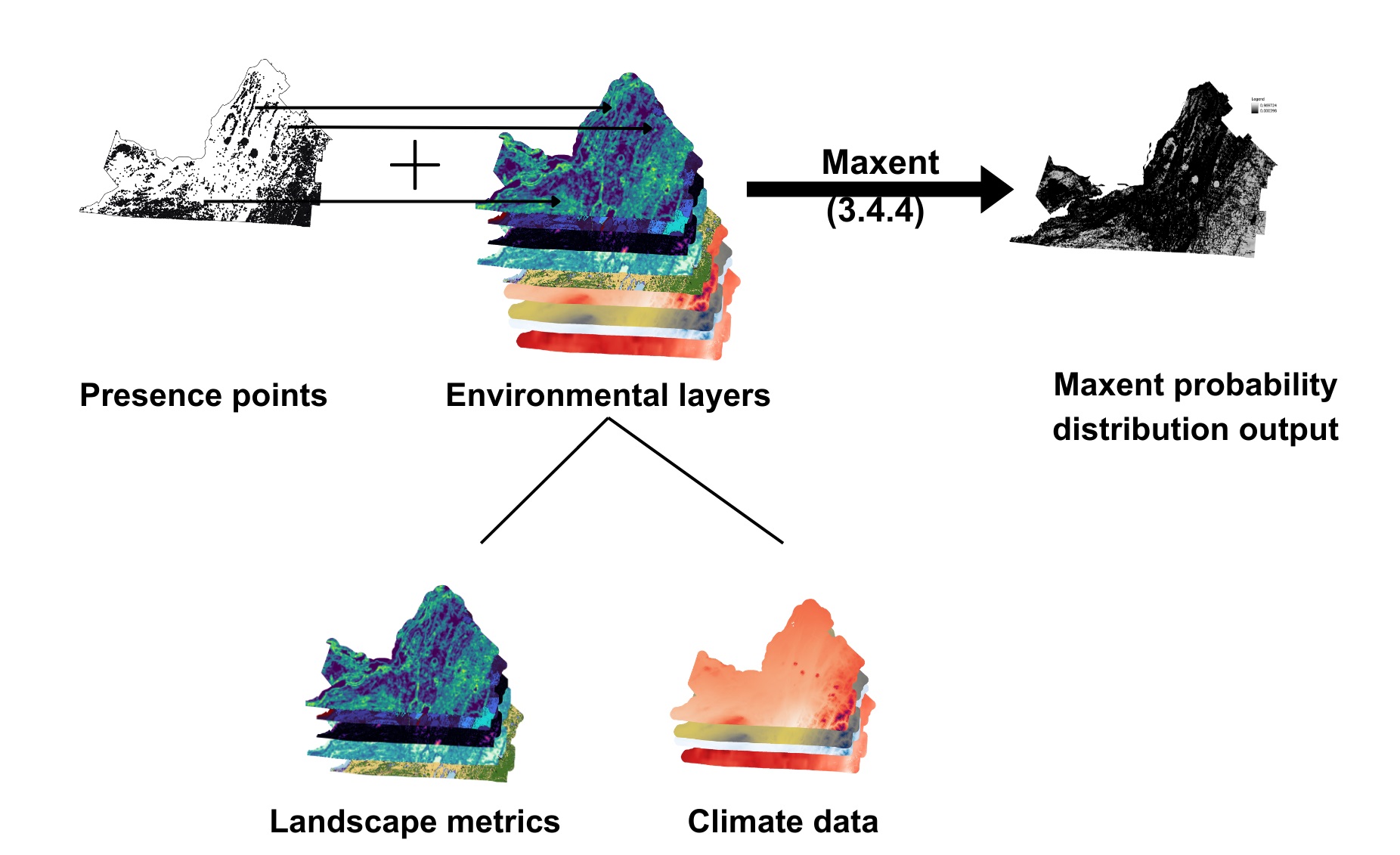

Supplement: Supplementary file 1 — Supplementary file1 (ZIP 14048 KB) [file 10980_2025_2121_MOESM1_ESM.zip › Supplementary_information_Destrempes/Image/Fig.S1.qgis.canva.jpg]

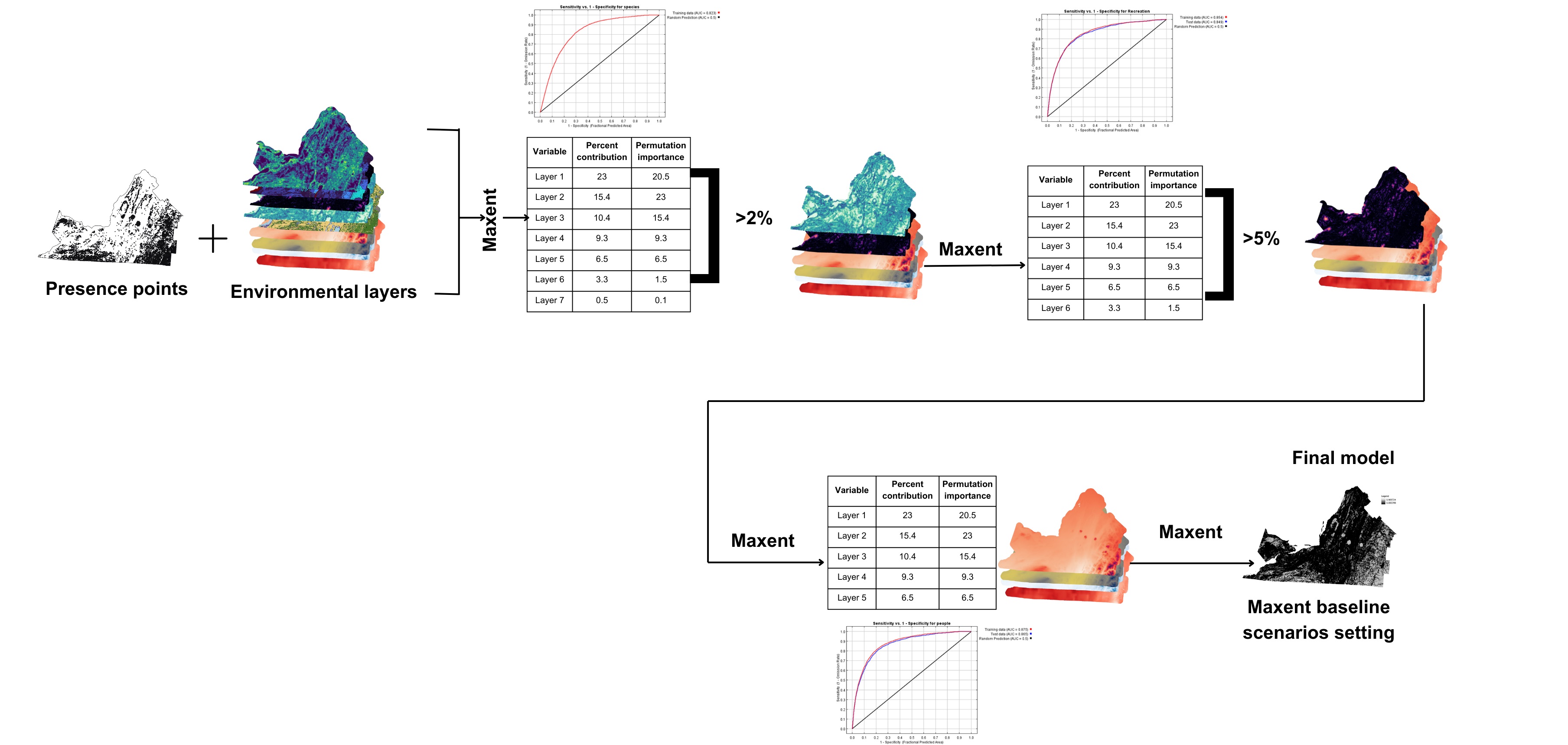

Supplement: Supplementary file 1 — Supplementary file1 (ZIP 14048 KB) [file 10980_2025_2121_MOESM1_ESM.zip › Supplementary_information_Destrempes/Image/Fig.S2.qgis.canva.jpg]

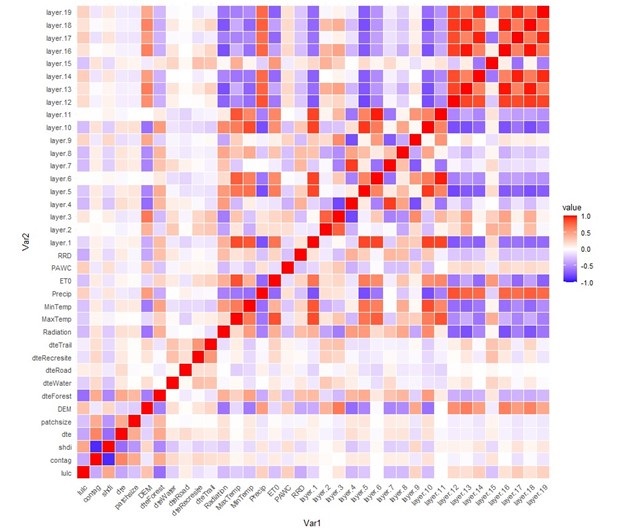

Supplement: Supplementary file 1 — Supplementary file1 (ZIP 14048 KB) [file 10980_2025_2121_MOESM1_ESM.zip › Supplementary_information_Destrempes/Image/Fig.S3_r.jpg]

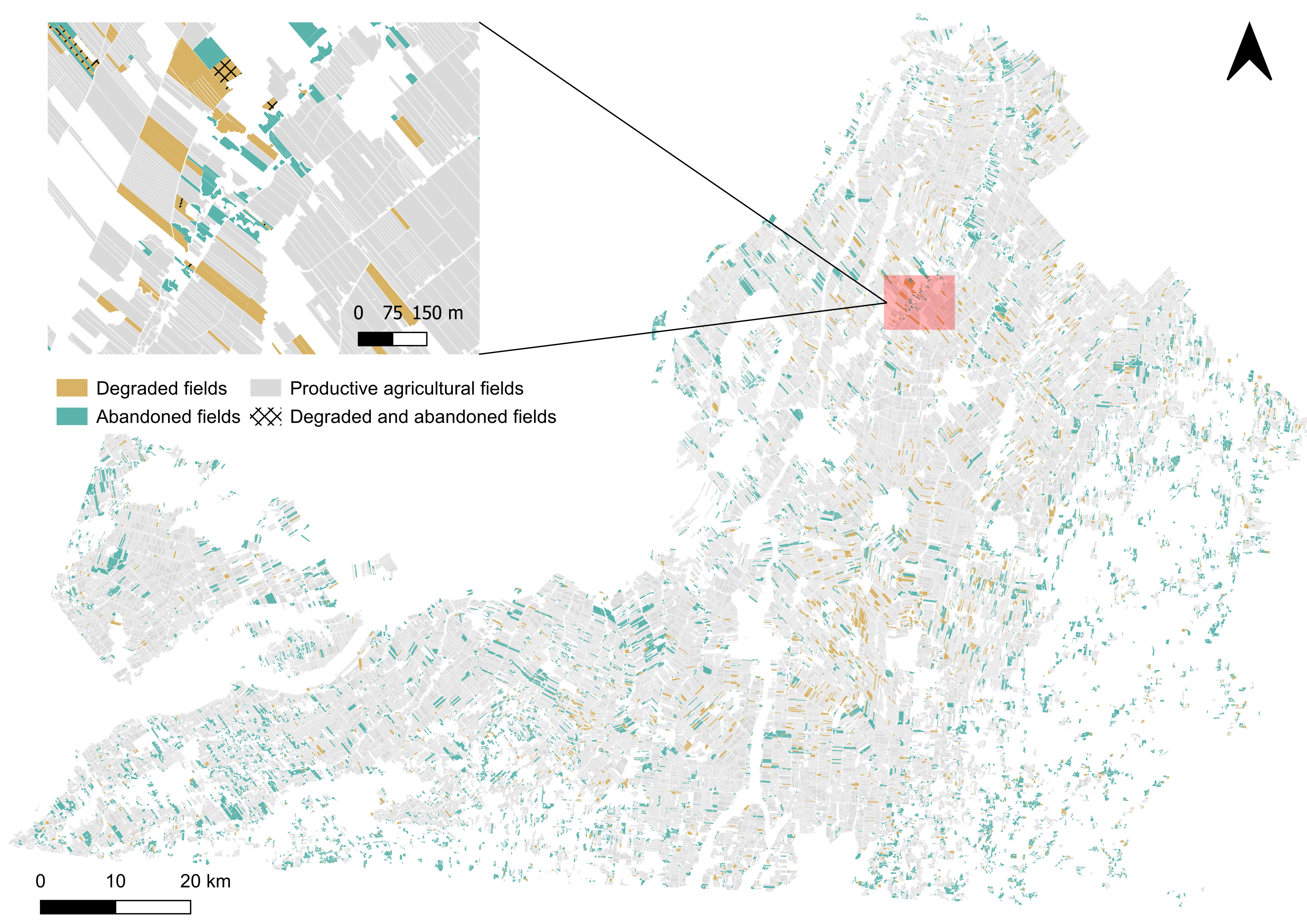

Supplement: Supplementary file 1 — Supplementary file1 (ZIP 14048 KB) [file 10980_2025_2121_MOESM1_ESM.zip › Supplementary_information_Destrempes/Image/Fig.S4_qgis.png]

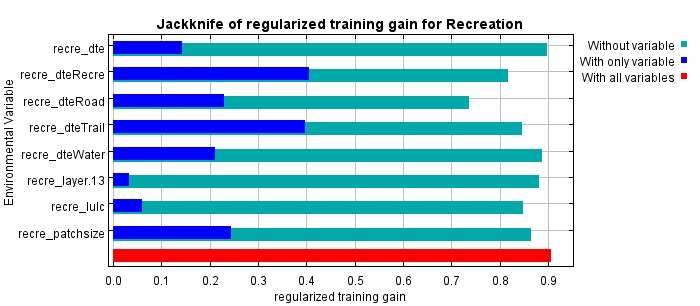

Supplement: Supplementary file 1 — Supplementary file1 (ZIP 14048 KB) [file 10980_2025_2121_MOESM1_ESM.zip › Supplementary_information_Destrempes/Image/Fig.S5.a.maxent.jpg]

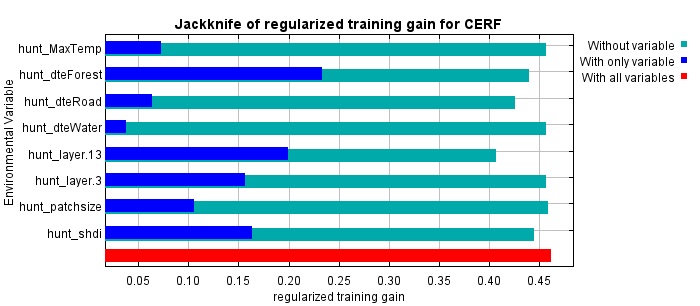

Supplement: Supplementary file 1 — Supplementary file1 (ZIP 14048 KB) [file 10980_2025_2121_MOESM1_ESM.zip › Supplementary_information_Destrempes/Image/Fig.S5.b.maxent.jpg]

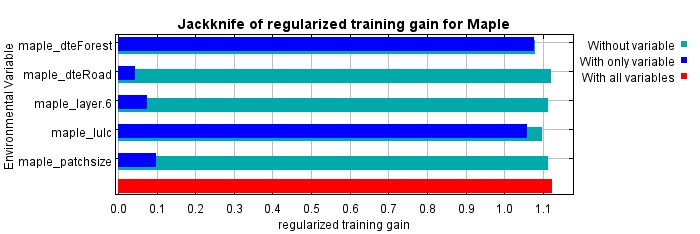

Supplement: Supplementary file 1 — Supplementary file1 (ZIP 14048 KB) [file 10980_2025_2121_MOESM1_ESM.zip › Supplementary_information_Destrempes/Image/Fig.S5.c.maxent.jpg]

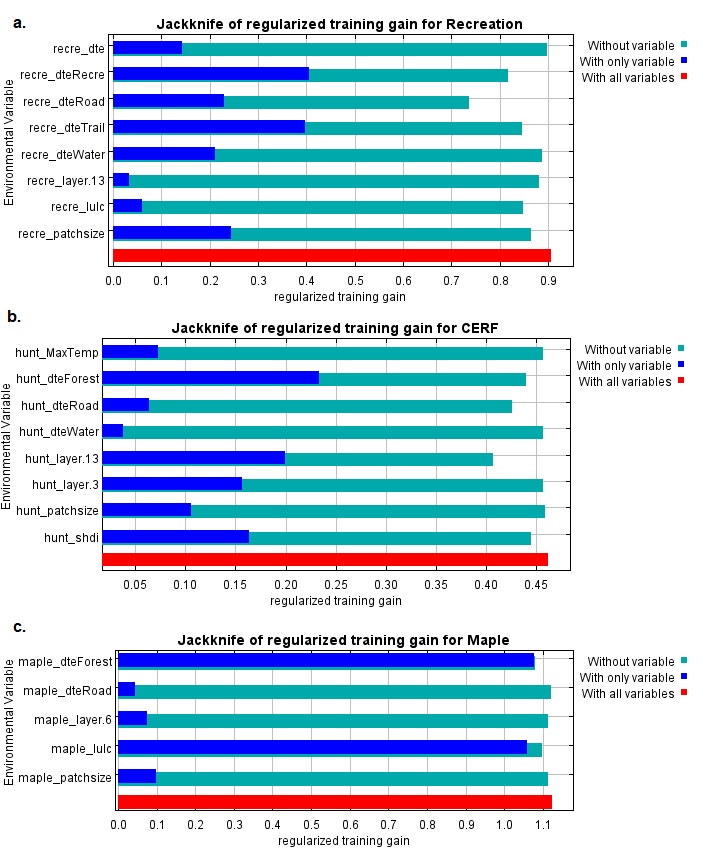

Supplement: Supplementary file 1 — Supplementary file1 (ZIP 14048 KB) [file 10980_2025_2121_MOESM1_ESM.zip › Supplementary_information_Destrempes/Image/Fig.S5.maxent.paint.jpg]

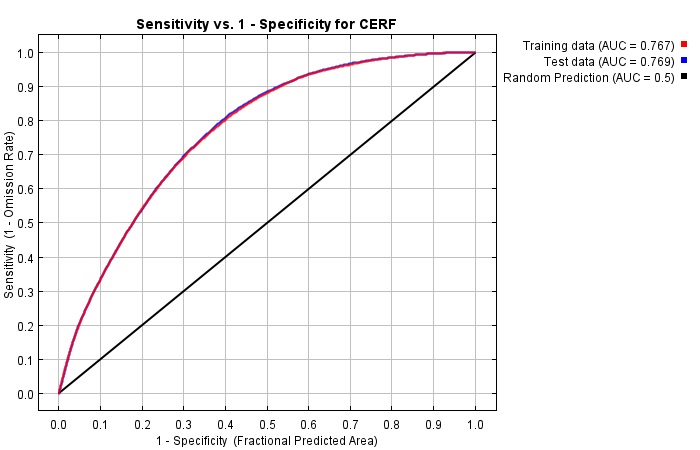

Supplement: Supplementary file 1 — Supplementary file1 (ZIP 14048 KB) [file 10980_2025_2121_MOESM1_ESM.zip › Supplementary_information_Destrempes/Image/Fig.S6.a.maxent.jpg]

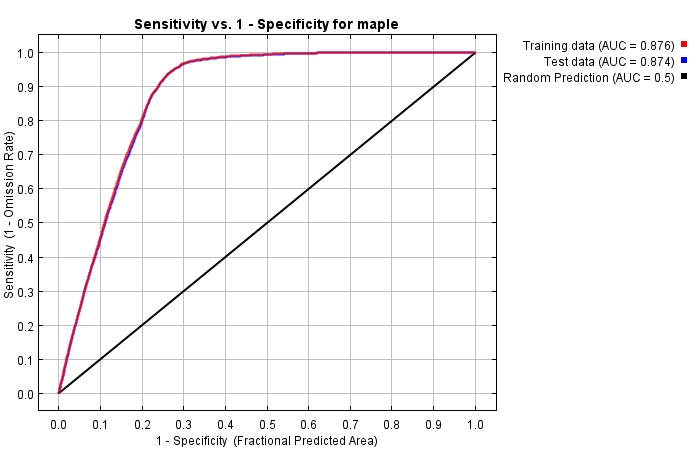

Supplement: Supplementary file 1 — Supplementary file1 (ZIP 14048 KB) [file 10980_2025_2121_MOESM1_ESM.zip › Supplementary_information_Destrempes/Image/Fig.S6.b.maxent.jpg]

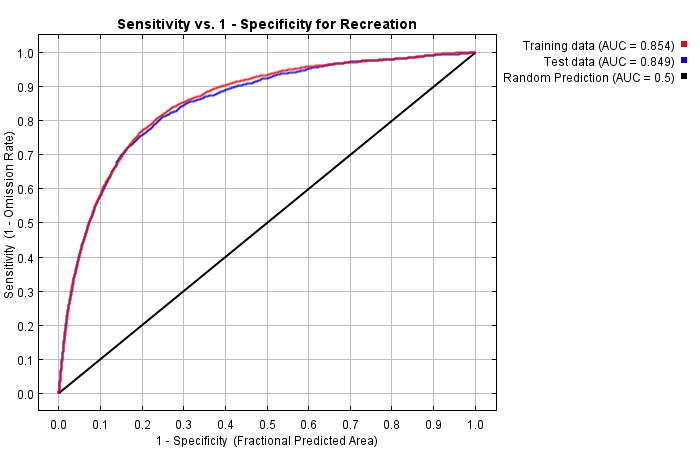

Supplement: Supplementary file 1 — Supplementary file1 (ZIP 14048 KB) [file 10980_2025_2121_MOESM1_ESM.zip › Supplementary_information_Destrempes/Image/Fig.S6.c.maxent.jpg]

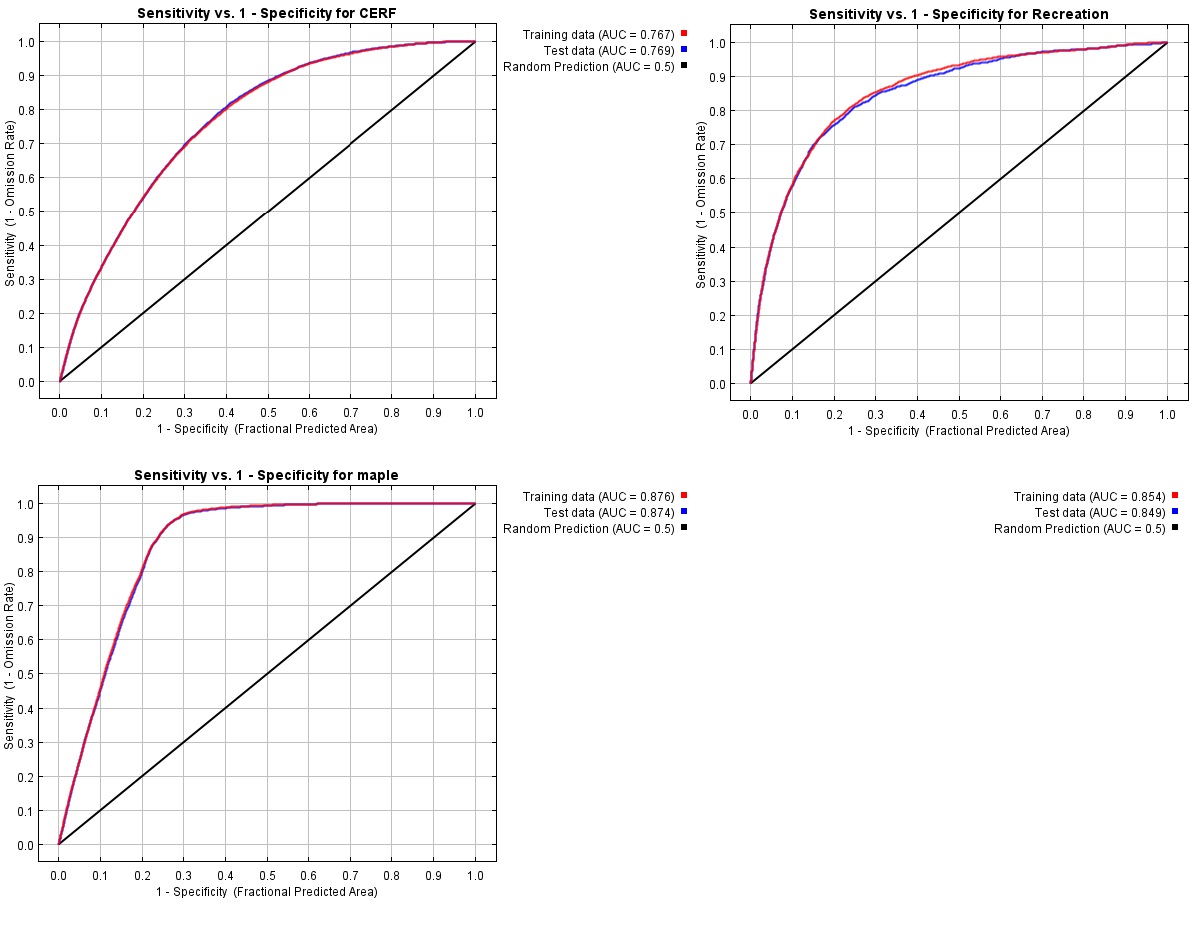

Supplement: Supplementary file 1 — Supplementary file1 (ZIP 14048 KB) [file 10980_2025_2121_MOESM1_ESM.zip › Supplementary_information_Destrempes/Image/Fig.S6.maxent.paint_V2.jpg]

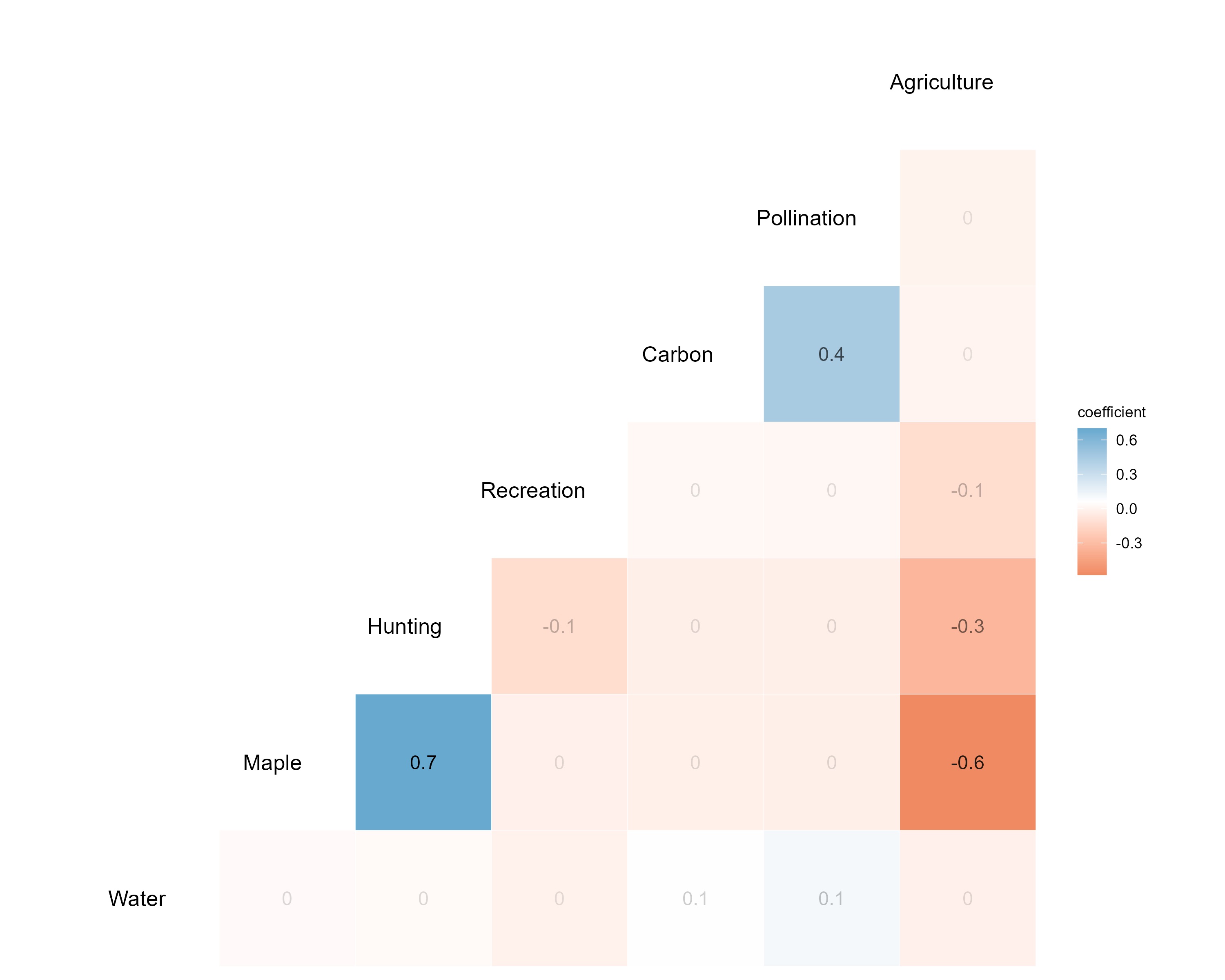

Supplement: Supplementary file 1 — Supplementary file1 (ZIP 14048 KB) [file 10980_2025_2121_MOESM1_ESM.zip › Supplementary_information_Destrempes/Image/Fig.S7_r.jpg]
